# Strengthening spatial reasoning: elucidating the attentional and neural mechanisms associated with mental rotation skill development

**DOI:** 10.1186/s41235-020-00211-y

**Published:** 2020-05-05

**Authors:** Katherine C. Moen, Melissa R. Beck, Stephanie M. Saltzmann, Tovah M. Cowan, Lauryn M. Burleigh, Leslie G. Butler, Jagannathan Ramanujam, Alex S. Cohen, Steven G. Greening

**Affiliations:** 1 Department of Psychology, Louisiana State University, 236 Audubon Hall, Baton Rouge, LA 70803 USA; 2 Department of Psychology, University of Nebraska at Kearney, Kearney, NE USA; 3 Department of Chemistry, Louisiana State University, Baton Rouge, LA USA; 4 Center for Computation and Technology, Louisiana State University, Baton Rouge, LA USA

**Keywords:** Spatial reasoning, Mental rotation, Encoding, Working memory, Eye-tracking, fMRI, Object representations, STEM education

## Abstract

Spatial reasoning is a critical skill in many everyday tasks and in science, technology, engineering, and mathematics disciplines. The current study examined how training on mental rotation (a spatial reasoning task) impacts the completeness of an encoded representation and the ability to rotate the representation. We used a multisession, multimethod design with an active control group to determine how mental rotation ability impacts performance for a trained stimulus category and an untrained stimulus category. Participants in the experimental group (*n* = 18) showed greater improvement than the active control group (*n* = 18) on the mental rotation tasks. The number of saccades between objects decreased and saccade amplitude increased after training, suggesting that participants in the experimental group encoded more of the object and possibly had more complete mental representations after training. Functional magnetic resonance imaging data revealed distinct neural activation associated with mental rotation, notably in the right motor cortex and right lateral occipital cortex. These brain areas are often associated with rotation and encoding complete representations, respectively. Furthermore, logistic regression revealed that activation in these brain regions during the post-training scan significantly predicted training group assignment. Overall, the current study suggests that effective mental rotation training protocols should aim to improve the encoding and manipulation of mental representations.

## Significance

In this study, we demonstrate that improvements in mental rotation performance are due in part to improved encoding of object representations and improved manipulation of these representations. Therefore, designing training protocols that target these cognitive processes may optimize the effectiveness of training and improve performance in tasks requiring spatial reasoning.

## Background

Mental rotation is a spatial reasoning task that is pervasive in everyday activities (e.g., driving, reading maps, filling the dishwasher, building Lego sets), and mental rotation performance is predictive of creativity, general intelligence, success in science, technology, engineering, and mathematics (STEM) disciplines (Wai, Lubinski, & Benbow, 2009), and success in several professions, including chemistry (Bodner & Guay, 1997; Harle & Towns, 2011), engineering (Samsudin, Rafi, & Hanif, 2011; Sorby, 2009), surgery (Stransky, Wilcox, & Dubrowski, 2010), and aviation (Dror, Kosslyn, & Waag, 1993). Understanding how to improve mental rotation is critical for STEM education. The number of U.S. citizens being successfully trained to perform STEM-related jobs does not meet the growing demand (U.S. Congress Joint Economic Committee, 2012). Nearly half of the students who begin a bachelor’s degree in STEM subjects do not complete the degree (Chen, 2013), and many students abandon STEM training due to underdeveloped skills (Higher Education Research Institute, 2010). Given ongoing debate about the effectiveness of “brain training” (see Simons et al., 2016, for review), it is critical to understand when and how training can be effective in improving mental rotation performance.

The current study combines measurements of eye movements and neural activity to better understand the extent to which mental rotation can be improved and the roles of core cognitive processes (e.g., encoding and transforming mental representations) in improving performance. Mental rotation is a spatial reasoning task in which participants must encode a representation of one object and then rotate that representation to judge if it can match another presented view of an object (Terlecki, Newcombe, & Little, 2008). Training on mental rotation tasks can lead to stable and transferable improvements in performance (see Uttal et al., 2013, for review), and mental rotation performance is associated with specific patterns of brain activity (Logie, Pernet, Buonocore, & Della Sala, 2011). However, it is not well understood which cognitive processes are involved in skill improvement. In the current study, we employed an optimal training method (see Simons et al., 2016) to examine the neurocognitive mechanisms associated with mental rotation improvement.

## Encoding and rotating during mental rotation

In a typical mental rotation task, the participant determines if two objects, presented side by side, are rotated versions of the same object. This requires at least two key components: (1) *encoding* a representation of the first fixated object and (2) *rotation* of this representation to determine if it matches the second object. Afterward, a comparison of the rotated mental image to this second object precipitates a decision (Larsen, 2014). Therefore, spatial reasoning improvement can occur by learning to encode a more complete representation and/or by learning to mentally rotate the encoded representation more effectively.

### Encoding

Mental rotation performance and efficiency improve when a more complete representation is encoded (Heil & Jansen-Osmann, 2008), and in particular when this representation includes multiple object parts and the spatial relationships between parts (Erdogan, Chen, Garcea, Mahon, & Jacobs, 2016). One way to measure the extent of encoding is with saccades. Saccades are eye movements between fixations, and the amplitude of a saccade is the distance between two consecutive fixations on an object. When this distance increases, it suggests that a larger area of the object is being processed and a more complete representation of the objects is being formed. For example, when looking at your friend, if your first fixation is on her forehead and then the second fixation is on her eye, this would be a short saccade and would suggest that a small area of your friend’s face was encoded during the first fixation. However, if the second fixation is on her chin, this would be a long saccade between consecutive fixations and would suggest that a larger area of your friend’s face was encoded during the first fixation. Encoding a more complete representation of an object is evidenced by a larger distance between successive fixations (i.e., saccade amplitude; Davitt, Cristino, Wong, & Leek, 2014; Irwin & Brockmole, 2000; Larsen, 2014) and by fewer saccades between objects (i.e., fewer encode–rotate–compare iterations; Larsen, 2014). Neurologically, lateral occipital cortex (LOC) activity has been shown to increase in response to encoding object parts and the spatial relationships among them (Erdogan et al., 2016), although it is also possible that LOC activity would decrease if encoding were to become more efficient. Therefore, if training leads to encoding more complete representations, we would expect to find increased saccade amplitude, fewer saccades between objects, and a change in LOC activity.

### Rotation

Mental rotation performance may improve not only because a more effective representation has been encoded but also because the process of mentally rotating the representation has improved. In mental rotation tasks, the difference between the two stimuli varies across trials in the angle of disparity (0–180 degrees), and response time (RT) increases as a function of the angle of disparity (Shepard & Metzler, 1971). This is taken as evidence that the participant is rotating a mental representation of the stimuli, because the further the stimuli needs to be rotated to match the other stimulus, the longer the RT should be (Shepard & Metzler, 1971). It is expected that the slope of the RT by angle of disparity function would decrease as mental rotation skills improve. In addition, greater activation in motor areas (e.g., primary motor, M1, and premotor areas) and regions of the visuospatial network (VSN; e.g., superior parietal lobe) is observed on mental rotation trials compared with nonrotation control trials, and this activation increases linearly as a function of angular disparity (Carpenter, Just, Keller, Eddy, & Thulborn, 1999; Leek & Johnston, 2009; Logie et al., 2011; Zacks, 2008; Zacks, Vettle, & Michelon, 2003). Therefore, as mental rotation skills improve, the RT slope may decrease, and activation in the VSN and motor areas could increase.

## Training in mental rotation skills

Although many studies have shown that practicing mental rotation can lead to an improvement in spatial reasoning skills (Cohen & Hegarty, 2007; Hoyek, Collet, Di Rienzo, De Almeida, & Guillot, 2014; Meneghetti, Borella, & Pazzaglia, 2016; Meneghetti, Cardillo, Mammarella, Caviola, & Borella, 2017; Rodán, Contreras, Elosúa, & Gimeno, 2016), these studies do not always use optimal methods for reliably detecting training effects (see Uttal et al., 2013, for review). Optimal methods include active control groups in which the control training task has levels of difficulty similar to those of the experimental task (Simons et al., 2016). Furthermore, it is important to use novel stimuli throughout training and at test to rule out improvements in performance due to familiarity with repeated stimuli (Tarr & Pinker, 1989). The majority of previous mental rotation training studies have used three-dimensional (3D) cubes similar to those used in the current study (Cohen & Hegarty, 2007; Meneghetti et al., 2017). Some studies have used 3D stimuli that more directly apply to everyday life, such as human anatomy (Hoyek et al., 2014) or a combination of 3D cubes and 2D drawings (Meneghetti et al., 2016; Rodán et al., 2016). However, previous studies do not specify if novel stimuli are used throughout training. Finally, it is unclear from previous research, but is important to determine, the extent to which training effects for mental rotation ability generalize to a category of stimuli that was not used during training (Meneghetti et al., 2017; Uttal et al., 2013; Wraga, Thompson, Alpert, & Kosslyn, 2003; Wright, Thompson, Ganis, Newcombe, & Kosslyn, 2008). Therefore, we include a mental rotation task pre- and post-training with a novel category of stimuli.

In the current study, we aimed to use an optimal training design to examine the effects of training on mental rotation performance for novel stimuli from the trained stimulus category (3D cubes) and for a novel category of stimuli (3D molecule ball-and-stick structures). Consistent with a lot of the previous research, which has focused on young adults as participants (Cohen & Hegarty, 2007; Meneghetti et al., 2017), and given the interest in applying these results to the training of STEM students, we recruited undergraduate science majors from introductory chemistry courses to participate in the study. We then randomly assigned matched pairs (on age, gender, and pre-training performance) to the training group or an active control group. The active control group performed a number estimation task that was similar to the mental rotation task in that it required the same or different judgment and varied in difficulty across trials (greater log difference between the two stimuli equals less difficulty), but the task did not require spatial reasoning skills (Park & Brannon, 2014). Importantly, and novel to previous studies, we used both eye-tracking and functional magnetic resonance imaging (fMRI) to evaluate changes in attention allocation and neural activity associated with improvements from training. This allowed us to determine the roles of (1) encoding more complete representations and (2) the ability to mentally rotate the representation in improved spatial reasoning ability.

## Method

### Participants

Sixty-seven undergraduate students enrolled in undergraduate introduction to chemistry courses (CHEM 1001 – Chemical Fundamentals, CHEM 1002 – Chemistry of Life and the Environment, CHEM 1201 – General Chemistry I, or CHEM 1202 – General Chemistry) began the study. Data from one participant was excluded because of excess movement during the pre-training fMRI. Data from two participants were excluded because of experimenter error during the pre-training eye-tracking tasks. Data from three participants were excluded because of nonremovable metallic material undisclosed at prescreening. Data from five participants were excluded because of below-chance accuracy on the pre-training behavioral measures. Twenty participants voluntarily withdrew from the study. Thirty-six participants (15 males; mean age, 18.97 years; SD, 1.36 years) participated in and completed the study. Our exclusion criteria included current prescription for medications to treat mental illness, formal diagnosis of a mental illness, claustrophobia, color vision deficiency, self-reported difficulty sitting still for long periods of time, and/or nonremovable metallic material anywhere in the body. On average, participants completed the study over 9.1 weeks. Participants were compensated $12 per hour. In addition, rewards and a bonus were used to encourage retention in the training program. A $100 reward was given to the top scorer across both conditions (total score across six training sessions), and $50 was given for the most improved (difference between training 1 accuracy and training 6 accuracy). There was also a random drawing for $50. This was done for each semester of data collection, so the rewards and random drawings were given at the end of Fall 2017, Spring 2018, and Summer 2018. Participants also received a $50 bonus for completing all ten sessions.

### Materials

#### Cubes

**Fig. 1:**
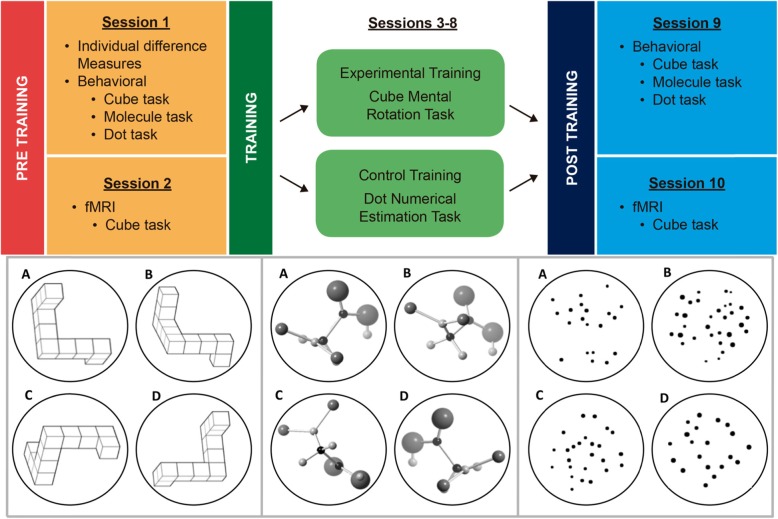
The current study used a multimethod, multisession training design (top panel). Participants were matched and assigned to the experimental group or active control training group. Participants were asked to make same/different judgments for three-dimensional cube arrangements (bottom left panel), three-dimensional chemical structures (bottom center panel), and random dot arrays (bottom right panel, control task). If presented with A and B, participants responded quickly and accurately because the angular disparity (40 degrees) is small (bottom left and center panels) or there is a large log difference (0.5) between the arrays (bottom right panel). When presented with A and C, participants responded more slowly and less accurately because of the larger angular disparity (120 degrees, bottom left and center panels) or because of the smaller log differences (0.2, bottom right panel). Object D in the bottom left and center panels represents a mirror image of object A (i.e., different object). Object D in the bottom right panel contains the same number of dots as object A (i.e., log difference of zero)

Each 3D block cube arrangement consisted of 9–11 individual white cubes on a black background (Fig. 1, bottom left). Each cube arrangement subtended 7.23 degrees of visual angle horizontally and 7.7 degrees of visual angle vertically. Sixteen unique arrangements, henceforth referred to as “3D cubes,” were used in each session (two pre-training sessions, six training sessions, and two post-training sessions) for a total of 160 unique 3D cubes. Therefore, a specific cube arrangement never repeated across sessions, and all cube arrangements in the post-training session had never been viewed during training. The cubes were arranged and rotated in a 3D space using MATLAB software (MathWorks, Natick, MA, USA) (Gee & Gissen, 2016). The 3D cubes were separately rotated along the *x*- and *y*-axes, and a version was created for every 10 degrees of rotation for a total of 72 different viewpoint versions of each 3D cube (36 *x*-axis, 36 *y*-axis). The 72 viewpoints were then mirror-imaged in PhotoShop software (Adobe, San Jose, CA, USA) to create 72 mirror-imaged viewpoints. Only 18–20 of these 144 viewpoints were used to create the pairs needed for a given mental rotation task, and each of these viewpoints was presented in only one trial. Viewpoint versions of each 3D cube were paired on the basis of degree of angular disparity between the two viewpoints in the pair, the trial type (same/different), and the rotation axis. To limit repetitions of the same stimulus across the trials, each stimulus was assigned only five angular disparities that could occur between the pairs (0, 40, 80, 120, 160, or 20, 60, 100, 140). Viewpoint versions of each 3D cube were presented on ten trials during a given session (two at each angular disparity: 0, 40, 80, 120, 160, or 20, 60, 100, 140; half same, half different; axis of rotation determined randomly for each pair).

#### Molecules

The molecular structures were ball-and-stick–type figures, which were modified versions of common chemical compounds available on ChemSpider (Fig. 1, bottom center). All chemical structures, henceforth referred to as “molecules,” had between 8 and 12 atoms and were modified using CrystalMaker 9.2 (CrystalMaker Software Ltd, Begbroke, UK). Exact structures of common chemical compounds were not used to ensure that participants had no existing knowledge of the specific structures. Each molecule arrangement fit within 7.23 degrees of visual angle horizontally and 7.7 degrees of visual angle vertically. The molecules were varying shades of gray and black to ensure that participants could distinguish the different parts, and they were presented on a gray background. Sixteen unique molecules were used during the pre- and post-training sessions for a total of 32 unique molecules. CrystalMaker 9.2 was used to create rotated versions of the molecules. The molecules were separately rotated along the *x*- and *y*-axes, and a version was created for every 10 degrees of rotation. Using the same procedure as the 3D cubes, different rotations of each molecule were paired on the basis of degree of angular disparity, trial type (same/different), and rotation axis.

#### Dots

The stimuli for the number estimation task (control training) were taken from Park and Brannon (2014). The stimuli were white dots (15–76) presented in a circular pattern on a black background (Fig. 1, bottom right). Dots were separated from one another by no less than four pixels. Each group of dots subtended 7.23 degrees of visual angle horizontally and 7.7 degrees of visual angle vertically.

### Procedure

The experiment consisted of ten sessions (Fig. 1, top panel). The first session consisted of behavioral measures of accuracy, RT, and eye movements during two different mental rotation tasks (cubes and molecules). The second pre-training session consisted of an fMRI brain scan while participants completed a mental rotation task (cubes). Participants were then matched in pairs based on sex, age, pre-training accuracy, and chemistry class; were assigned to the control training (number estimation task) or experimental training (cube training task); and completed six training sessions over 2–3 weeks (Table 1). After training, participants completed two post-training sessions (one behavioral session with eye-tracking and one fMRI session), which were identical to the two pre-training sessions, with the exception that participants did not complete questionnaires during the post-training sessions. Below we provide a detailed description of each phase of the experimental protocol (Fig. 1, top panel), which included two pre-training days (sessions 1 and 2), six training days (experimental training or control training; sessions 3–8), and two post-training days (sessions 9 and 10).

**Table 1:** No differences in matched pairs

	Experimental group Mean (SD)	Control group Mean (SD)	*t*	*p* Value	Mean difference (95% CI)	BF_10_
Age	19.06 (1.70)	18.89 (1.02)	0.36	.72	0.17 (−0.78, 1.11)	< 1
GPA	3.34 (0.80)	3.42 (0.60)	0.33	.74	−0.08 (− 0.57, 0.41)	< 1
Study duration (weeks)	9.24 (2.52)	8.83 (3.39)	0.41	.68	0.41 (−1.61, 2.44)	< 1

*BF*_*10*_ Bayes factor, *CI* confidence interval, *GPA* grade point average

### Pre-training day 1

During day 1 of the pre-training session, participants completed several individual difference questionnaires, a measure of math ability, and two mental rotation tasks (cubes and molecules). The questionnaires and math measures are outside the scope of the current paper and were not impacted by training condition.

#### Mental rotation tasks

Participants completed two mental rotation tasks in counterbalanced order, one with 3D cubes and one with 3D ball-and-stick molecules. Administration of the stimuli and collection of accuracy, RT, and eye movements for each task were completed using the EyeLink 1000+ desk-mounted eye-tracker and software (SR Research, Ottawa, ON, Canada). The only difference in the methodology between the two tasks was the type of stimuli presented: cubes or molecules.

Before beginning the test trials, participants completed two practice trials (one same trial and one different trial, both at 100 degrees of angular disparity) and received feedback after each practice trial to ensure they understood the task. A 9-point eye-tracking calibration was completed after the practice trials. Participants viewed two 3D cubes, one on each side of the screen, with 6.43 degrees of visual angle between the cubes, during each trial. The angular disparity between the two stimuli ranged from 0 to 180 degrees of rotation in steps of 20 degrees along the *x*- or *y*-axis. Stimulus pairs were either rotated versions of the same object (same trials) or rotated versions of mirror-image objects (different trials). Participants were instructed to compare the two objects and respond via button box whether the two objects were rotated versions of the same object or different objects. Participants had 8 s to respond to each object pair and completed 160 trials, with 16 at each angular disparity (8 with the same object and 8 with the mirrored version of the object) presented in a random order. All participants completed 160 trials. Feedback was only provided during the practice trials. There was no feedback provided for the test trials.

### Pre-training day 2

The second pre-training session consisted of an fMRI brain scan while participants completed a mental rotation task with the cube stimuli.

#### MRI acquisition

Imaging data were collected using a Discovery MR750w 3.0-T system (GE Healthcare, Chicago, IL, USA) with a 32-channel head coil (MR Instruments, Minneapolis, MN, USA) at Pennington Biomedical Research Center, Baton Rouge, LA, USA. T1-weighted structural images were acquired using a 3D fast spoiled gradient-echo sequence (repetition time [TR] = 8.7 ms, echo time [TE] = 3.8 ms, flip angle = 8 degrees, 256 × 256 matrix, phase encoding direction anterior to posterior, field of view [FOV] = 25.6 cm). One hundred eighty sagittal slices covering the entire brain were acquired in sequential order, producing a voxel resolution of 1 mm isotropic. T2*-weighted functional scans were acquired using gradient echo echo-planar imaging (echo planar imaging; TR = 2000 ms, TE = 25 ms, flip angle = 90 degrees, 64 × 64 matrix, phase encoding direction anterior to posterior, FOV = 22.4 cm). Thirty-six axial slices covering the whole brain were acquired with a voxel resolution of 3.5 mm isotropic with no gap. Slices were acquired in interleaved ascending order. Each functional scan began with three dummy volumes to account for equilibrium effects, and these dummy volumes were discarded from the analyses during preprocessing. Each run of the mental rotation task contained 168 volumes. Participants also completed a basic mental imagery task (136 volumes) and resting state scan (150 volumes), neither of which is presented in the current study.

#### Mental rotation task

Participants were cleared to enter and were placed inside the scanner. A mirror was attached to the head coil so that the experimental task was visible to the participants. The current study used a blocked design with five runs. Each run consisted of six blocks (three rotation, three control) randomized throughout the run. During control blocks, the 3D arrangements were not rotated (zero degrees of angular disparity); thus, the blocks were either identical (same trial) or mirror images of each other (different trial). For the rotated trials, the 3D cube arrangements differed by 60, 100, or 140 degrees of angular disparity along the *x*- or *y*-axis. Each block was 30 s long, separated by a 20-s fixation. Each run began and ended with a 20-s fixation. Thus, each run was approximately 5.5 min long. A given trial began with a fixation cross in the center of the screen for 20 s followed by pairs of 3D cube arrangements. The cube arrangements were present on the screen for a maximum of 8 s, with a participant response ending the trial. This was to give the participants enough time to mentally rotate the object and make a decision. Participants were instructed to respond as quickly and accurately as possible, regardless of whether the two arrangements were rotated versions of the same arrangement or two different arrangements. Responses were recorded with a button box. There was no feedback provided during the mental rotation task in the scanner.

### Experimental training

Training consisted of six sessions over 2–3 weeks (average, 2.8 weeks). Each training session lasted approximately 45 min. Each participant was matched to another participant with the closest age, grade point average, and pre-training accuracy, as well as with the same sex and in the same chemistry course (Table 1). Matched pairs of participants were randomly assigned to the experimental training or control training condition.

The experimental training task was nearly identical to the pre-training cubes task, but a new set of 3D cubes was used in each training session to control for memory of the stimuli. Participants received feedback (“correct” or “incorrect”) and received points when they correctly identified two 3D cubes as being the same or different for all of the training trials. Point values ranged from 10 to 100 for correct answers, and more points were awarded for more difficult responses (e.g., correctly responding to objects with zero degrees of angular disparity resulted in 10 points, whereas 180 degrees of angular disparity resulted in 100 points). Zero points were awarded for incorrect trials. Participants completed a total of 160 trials per training session, regardless of accuracy.

### Control training

The control training was a number estimation task based on Park and Brannon (2014). Number estimation tasks have previously been associated with improved mathematical performance but are not thought to require spatial reasoning skills. Furthermore, the task required a same/different judgment and varied in difficulty across trials. Participants were presented with two random dot arrays containing 15–76 dots of varying sizes and were instructed to respond if the two dot arrays contained the same number of dots or a different number of dots. Participants had 8 s to respond to each pair, thus limiting the selection of a strategy of simply counting the number of dots. Difficulty was manipulated by altering the log differences between the number of dots in the two dot arrays (0.1, 0.2, 0.3, 0.4, 0.5, 0.6, 0.7, 0.8, 0.9, or 1.0). This manipulation allowed for ten levels of difficulty, similar to how the 10 degrees of rotation allowed for ten levels of difficulty in the experimental training task. Participants received feedback and points when they correctly identified whether the two random dot arrays contained the same or a different number of dots. Point values ranged from 10 to 100 points, and more points were awarded for more difficult responses (i.e., the smaller the log difference between the two dot arrays, the more difficult the trial, and the larger the number of points awarded for correct answers). Zero points were awarded for incorrect trials. Participants completed a total of 80 trials with zero log difference (same trials) and 8 trials at each of the 10 log differences (different trials), for a total of 160 trials per training session, regardless of accuracy.

### Post-training day 1

The first post-training session consisted of the same math test and two eye-tracking mental rotation tasks as in the pre-training. Participants were presented with new 3D cubes and molecules to control for memory of the stimuli.

### Post-training day 2

The second post-training session was identical to the pre-training day 2 fMRI task but also used a new set of 3D cubes to control for memory of the stimuli.

### Statistical analysis

Analysis of variance (ANOVA) and follow-up *t* tests were performed using Bayesian analyses and traditional null hypothesis testing. Bayes factors (BFs) are reported for all effects, regardless of *p* value. BFs greater than 1 are considered evidence for the alternative hypothesis (BF_10_), whereas BFs less than 1 are considered evidence for the null hypothesis (BF_01_). We subscribed to the standard support for the alternate hypothesis used in JASP open-source software, with a BF of 3 or less considered ambiguous evidence.

To examine the effects of training, we conducted 2 (training condition: experimental, control) × 2 (session: pre-training, post-training) mixed factors ANOVA for each dependent variable during the 3D cube and 3D molecule mental rotation tasks separately. Session was a within-subject factor, and training was a between-subjects factor. If there were any significant main effects or interactions, we followed up the analysis with paired sample *t* tests comparing the dependent variables at pre- and post-training for each training condition separately. We also conducted independent sample *t* tests to compare the two training conditions pre- and post-training. To summarize, for each dependent variable, we conducted one ANOVA and four *t* tests. Sex differences are often documented in mental rotation research (Geary, Saults, Liu, & Hoard, 2000). We added sex as a factor in every analysis, and it was never involved in a significant interaction, nor was there ever a significant main effect of sex (*p*_s_ > .07).

After data collection was completed, we became aware that four molecular images were not chiral (mirror-imaged) and “different” molecules could be rotated to match one another. In order to account for this issue, trials with these four molecules were excluded from the analyses. Twenty trials were excluded for each participant at both pre- and post-training. Pre-training, the 20 trials were from rotations 20, 60, 100, 140, and 180 (four trials at each of the five rotations; half same trials, half different trials). Post-training, the 20 trials were from rotations 0, 40, 80, 120, and 160 (four trials at each of the five rotations; half same trials, half different trials).

#### fMRI preprocessing and whole-brain univariate analyses

fMRI data were analyzed using FEAT (FMRI Expert Analysis Tool) version 6.0, part of FSL (FMRIB Software Library, Oxford, UK; www.fmrib.ox.ac.uk/fsl). Registration to participant structural and standard space images was carried out using FLIRT (FMRIB Linear Image Registration Tool, Oxford, UK) (Jenkinson, Bannister, Brady, & Smith, 2002; Jenkinson & Smith, 2001). Prestatistics processing applied included motion correction using MCFLIRT (Jenkinson et al., 2002), slice-timing correction using Fourier space time-series phase-shifting, nonbrain removal using the Brain Extraction Tool (Smith, 2002), spatial smoothing using a gaussian kernel of full width at half-maximum 7 mm, grand mean intensity normalization by a single multiplicative factor, and high-pass temporal filtering (gaussian weighted least squares straight line fitting with sigma = 50.0 s). The time-series modeling was carried out using FILM with local autocorrelation correction (Woolrich, Ripley, Brady, & Smith, 2001).

At the single-subject level, each run was modeled separately. A double-gamma hemodynamic response function convolution was used on each of the conditions of interest/explanatory variables (i.e., rotation blocks and control blocks) inputted in custom (three-column format) basic shape. Temporal derivatives of each condition of interest were added. Several nuisance regressors were added, including six original motion parameters, extended motion parameters, and framewise displacement = 0.9 mm motion censoring (Siegel et al., 2014) using the fsl_motion_outliers function. A second-level analysis was performed to average over contrast estimates from the first-level analysis for each experimental phase (e.g., rotation blocks and control blocks) for each participant. These analyses were carried out using a fixed effects model in FLAME (FMRIB’s Local Analysis of Mixed Effects), with the random effects variance forced to zero (Beckmann, Jenkinson, & Smith, 2003; Woolrich, Behrens, Beckmann, Jenkinson, & Smith, 2004). Group-level analyses were carried out using FLAME stage 1 (Beckmann et al., 2003; Woolrich et al., 2004). The resulting z (gaussianized T/F) statistic images were thresholded using clusters determined by z > 2.3 and a cluster significance threshold of *p* = 0.05 (Worsley, 2001).

A second first-level analysis was also carried out, which modeled the conditions in an event-related fashion (rather than a blocked design). This analysis included eight regressors of interest associated with each angular disparity (0, 60, 100, 140) and whether the trial involved a pair of stimuli that were identical (same) or mirror images (different). The purpose of this model was to assess how brain activation changed as a result of angular disparity change.

## Results

### Behavioral effects of training

#### Accuracy: cubes task

**Fig. 2:**
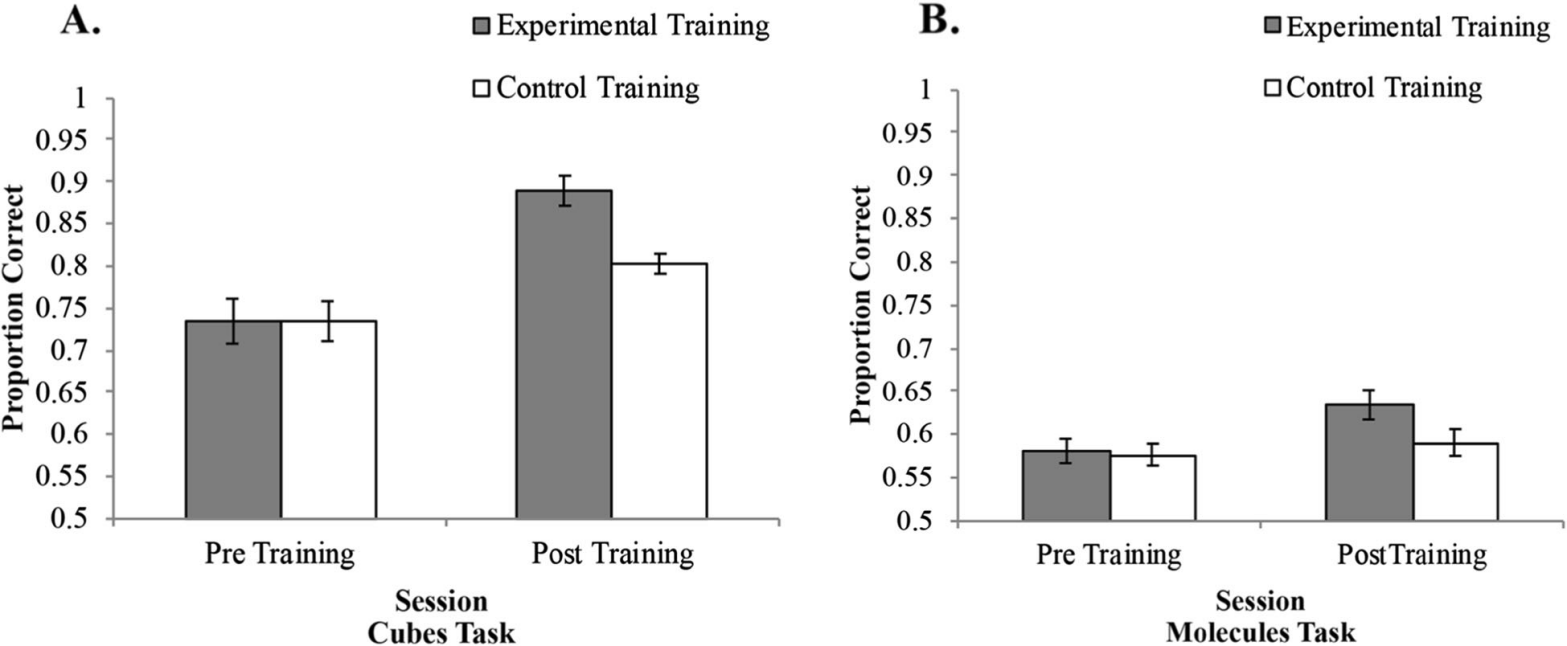
Before training, the training groups did not differ in accuracy on the cubes task (**a**) or the molecules task (**b**). After training, both training conditions improved on the cubes task (**a**), but the experimental group improved significantly more than the control group. Only the experimental group improved on the molecules task (**b**)

There was a significant main effect of session, *F*(1,34) = 131.83, *p* < .001, *η*^*2*^_*p*_ = .80, BF_10_ >  1000, in that accuracy was overall higher after training than before training (Fig. 2a). There was no main effect of training condition, *F*(1,34) = 1.94, *p* = .17, *η*^*2*^_*p*_ = .05, BF_01_ = 0.66. Importantly, there was a significant interaction between session and training condition, *F*(1,34) = 18.72, *p* < .001, *η*^*2*^_*p*_ = .36, BF_10_ >  1000. Accuracy increased pre- to post-training for both the experimental, *t*(17) = 10.15, *p* < .001, BF_10_ >  1000, and control groups, *t*(17) = 5.71, *p* < .001, BF_10_ = 947. Furthermore, there was no significant difference between the two training groups pre-training, *t*(34) = 0.03, *p* = .97, BF_01_ = 0.32, but post-training, the experimental group had significantly higher accuracy than the control group, *t*(34) = 2.97, *p* = .005, BF_10_ >  1000. Overall, these results suggest that the mental rotation training was effective in improving mental rotation accuracy for novel stimuli from the trained category.

#### Accuracy: molecules task

There was a significant main effect of session, *F*(1,34) = 9.91, *p* = .003, *η*^*2*^_*p*_ = .23, BF_10_ = 9.28, in that accuracy increased after training (Fig. 2b). There was no significant main effect of training condition, *F*(1,34) = 1.62, *p* = .21, *η*^*2*^_*p*_ = .05, BF_01_ = 0.60, nor was there an interaction between session and training condition, *F*(1,34) = 3.77, *p* = .061, *η*^*2*^_*p*_ = .10, BF_10_ = 1.47. Results revealed that the control training had no impact on accuracy on the molecules task, *t*(17) = 0.91, *p* = .38, BF_01_ = 0.35, but the experimental training significantly improved accuracy after training, *t*(17) = 3.41, *p* = .003, BF_01_ = 13.41. There were no differences in accuracy for the two training conditions pre-training, *t*(34) = 0.12, *p* = .91, BF_01_ = 0.32, or post-training, *t*(34) = 1.93, *p* = .06, BF_10_ = 1.33. Overall, these results suggest that repeated exposure to a mental rotation task with simple 3D cubes leads to a small improvement in mental rotation ability for a nontrained category of stimuli (3D molecules).

#### Response time: cubes task

**Fig. 3:**
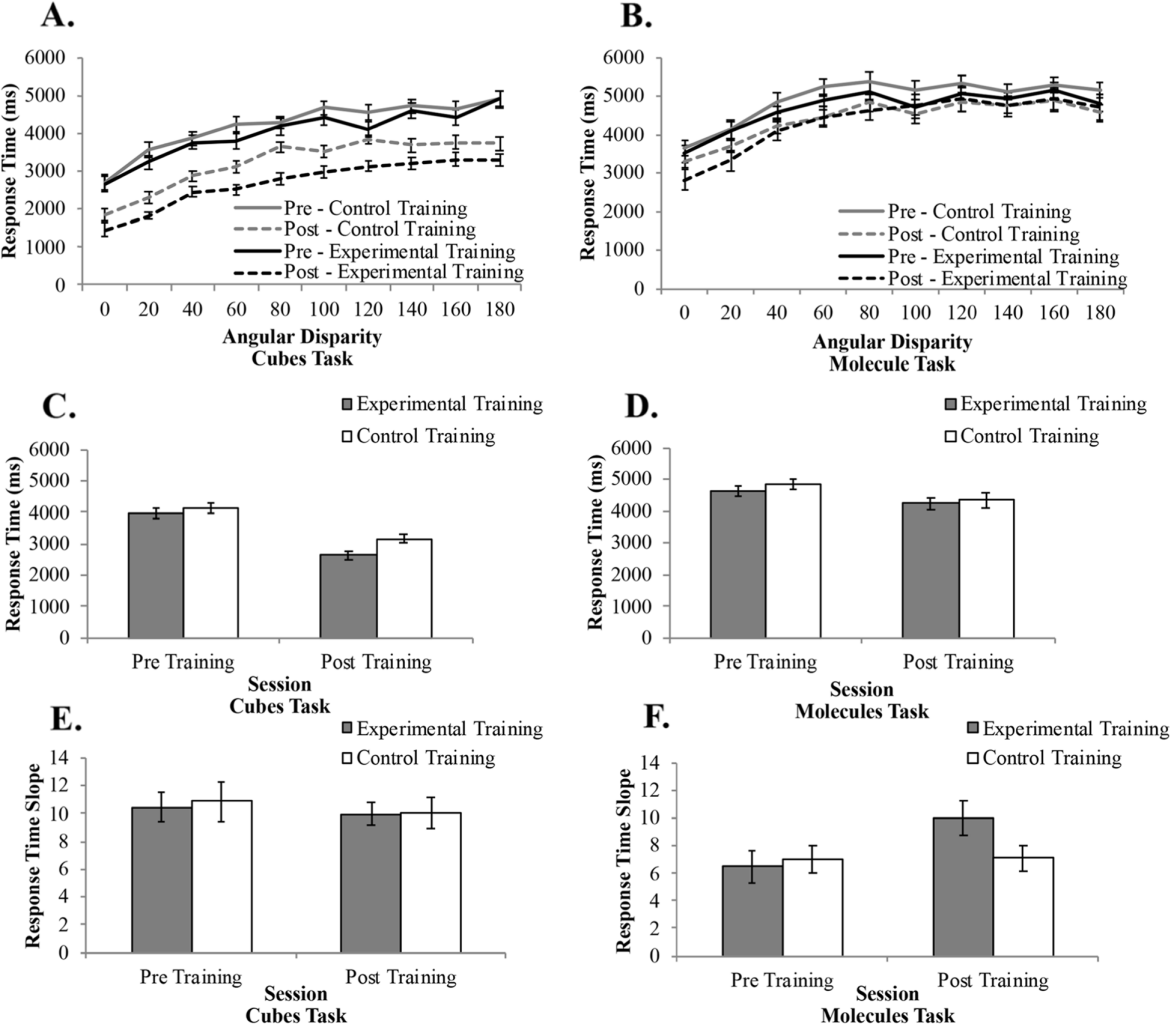
Response time increased with angular disparity for both the cubes (**a**) and molecules (**b**) tasks. Response time decreased after training for the cubes task (**c**) and the molecules task (**d**). Training did not impact the response time slope for the cubes task (**e**), but the experimental group had a significant increase in slope for the molecules task after training (**f**)

There was a linear relationship between RT and angular disparity (Fig. 3a), as is typically observed in mental rotation studies. However, angle of rotation never significantly interacted with training condition or session in an omnibus ANOVA; thus, angular disparity was dropped from the remaining analyses. There was a significant main effect of session, *F*(1,34) = 83.61, *p* < .001, *η*^*2*^_*p*_ = .71, BF_10_ >  1000, in that RTs were faster after training than before training (Fig. 3a). There was also a significant main effect of training condition, *F*(1,34) = 4.40, *p* = .043, *η*^*2*^_*p*_ = .12, BF_01_ = 0.72, in that the experimental training condition was faster overall than the control training condition. There was no significant interaction, *F*(1,34) = 1.68, *p* = .204, *η*^*2*^_*p*_ = .05, BF_01_ = 0.49. RT decreased pre- to post-training for both the experimental group, *t*(17) = 6.86, *p* < .001, BF_10_ >  1000, and the control group, *t*(17) = 6.04, *p* < .001, BF_10_ >  1000. There was no significant difference between the two training groups before training, *t*(34) = 0.83, *p* = .41, BF_01_ = 0.421, but after training, the experimental group had significantly faster RTs than the control group, *t*(34) = 2.79, *p* = .009, BF_10_ = 5.673. These results are in line with the accuracy data in that the experimental group had the greatest improvement after training.

In order to determine if participants were able to more efficiently rotate objects as a result of training, we further analyzed RT by calculating the slope of RT across the ten angular disparities. The results revealed no main effect of session (Fig. 3e), *F*(1,34) = 0.81, *p* = .37, *η*^*2*^_*p*_ = .02, BF_01_ = 0.31, or training condition, *F*(1,34) = 0.04, *p* = .85, *η*^*2*^_*p*_ = .001, BF_01_ = 0.31, and no interaction, *F*(1,34) = 0.08, *p* = .78, *η*^*2*^_*p*_ = .002, BF_01_ = 0.44.

#### Response time: molecules task

There was a linear relationship between RT and angular disparity (Fig. 3b), as is typically observed in mental rotation studies. However, angle of rotation never significantly interacted with training condition or session in an omnibus ANOVA; thus, angular disparity was dropped from the analyses. The results revealed a significant main effect of session, *F*(1,34) = 9.99, *p* = .003, *η*^*2*^_*p*_ = .23, BF_10_ = 13.17, in that RT decreased after training (Fig. 3b). There was no significant main effect of training condition, *F*(1,34) = 0.54, *p* = .47, *η*^*2*^_*p*_ = .02, BF_01_ = 0.41, nor was there a significant interaction, *F*(1,34) = 0.02, *p* = .66, *η*^*2*^_*p*_ = .006, BF_01_ = 0.29. The results revealed that the control training decreased RTs on the molecules task, *t*(17) = 2.42, *p* = .03, BF_01_ = 2.35, but the experimental training did not impact RT, *t*(17) = 2.03, *p* = .06, BF_01_ = 1.29. There were no differences in RT between the two training conditions pre-training, *t*(34) = 0.98, *p* = .34, BF_01_ = 0.34, or post-training, *t*(34) = 0.35, *p* = .73, BF_01_ = 0.47. Overall, the relationship between training condition and RT was ambiguous and suggested that mental rotation training may not impact RT for the molecules task.

As with the cubes task, we analyzed the slope of RT across the ten angular disparities. The results revealed no main effect of session, *F*(1,34) = 3.69, *p* = .06, *η*^*2*^_*p*_ = .10, BF_01_ = 0.91, or training condition, *F*(1,34) = 0.93, *p* = .34, *η*^*2*^_*p*_ = .03, BF_01_ = 0.50, but there was a significant interaction (Fig. 3f), *F*(1,34) = 5.87, *p* = .02, *η*^*2*^_*p*_ = .15, BF_10_ = 3.89. To examine the interaction, we conducted follow-up paired samples *t* tests to compare the slope before and after training for the two training conditions separately. The results revealed that the control training had no impact on RT slope on the molecules task, *t*(17) = 0.38, *p* = .71, BF_01_ = 0.26, but the experimental training had a significant increase in slope after training, *t*(17) = 2.88, *p* = .01, BF_10_ = 5.14. We further conducted separate independent samples *t* tests comparing the control group with the experimental group pre-training and post-training. The results revealed no differences in RT slope for the two training conditions pre-training, *t*(34) = 0.34, *p* = .74, BF_01_ = 0.34, or post-training, *t*(34) = 1.98, *p* = .06, BF_10_ = 1.42.

#### Behavioral results summary

The behavioral data above suggest that mental rotation training leads to increased accuracy and decreased RT for novel stimuli in a 3D cube task, and this training showed some, although weak, evidence of transfer to a nontrained category (3D molecules). It is important to note that these generalization effects were found for accuracy and not RT and that they were small and not supported by a significant interaction. Nevertheless, follow-up *t* tests and BFs suggested that the experimental training group had increased accuracy on the molecules task following training. Finally, these data do not indicate whether the source of the improvement is due to a more complete representational encoding or an enhanced rotation process. Thus, the current study also used eye-tracking and fMRI in order to determine the attentional and neurological sources of the improvement in mental rotation training.

### Oculomotor effects of training

#### Saccade amplitude: cubes task

**Fig. 4:**
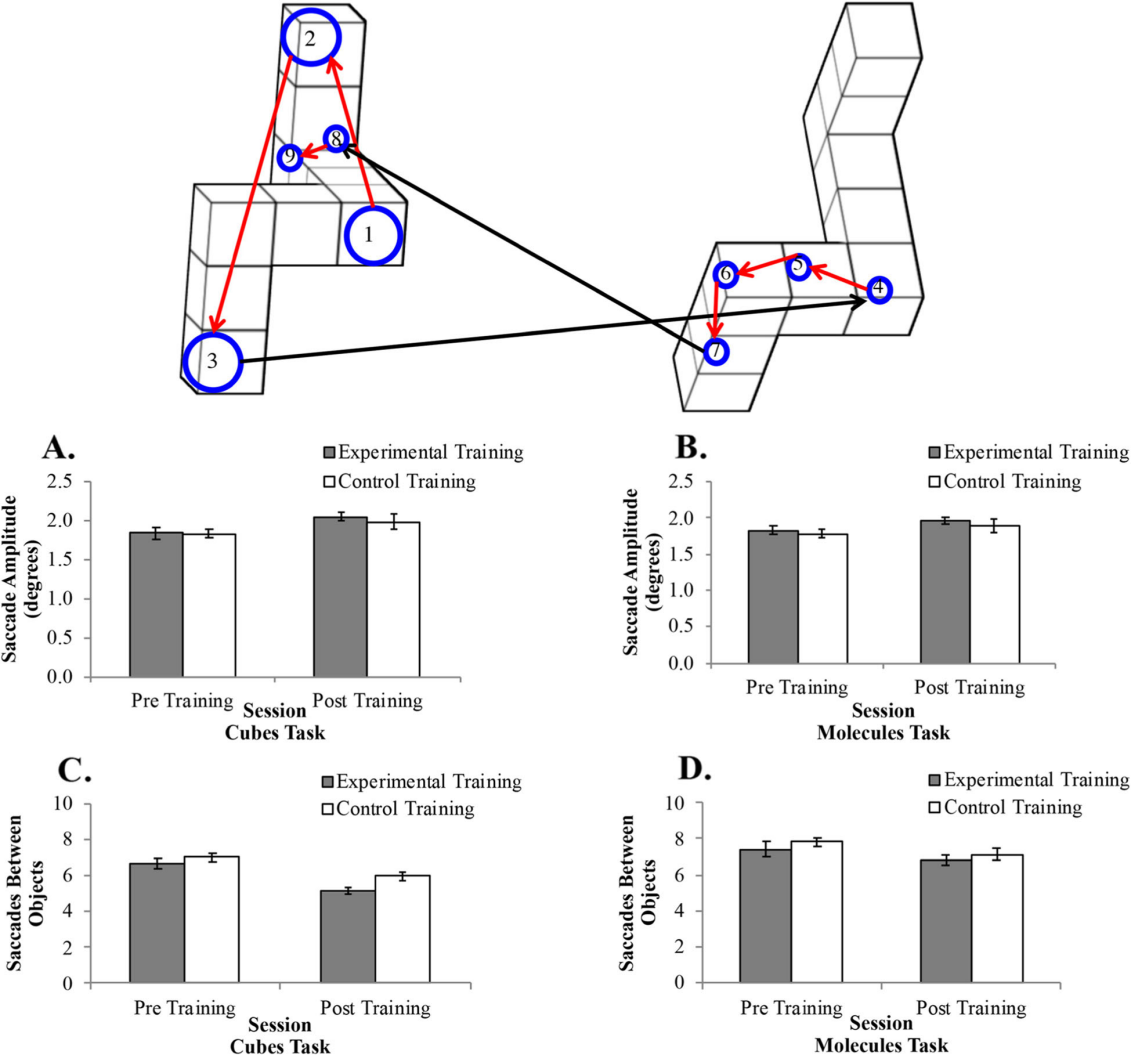
The top panel represents a sample trial during the pre-training session of one participant. Blue circles represent fixations, and the numbers inside circles represent fixation order. The diameter of the circle represents the duration of the fixation. The red arrows from one circle to the next represent saccades within an object. Long black arrows represent switches between objects. This participant initially used a global strategy (fixations 1–3; long fixations, long saccades) and switched to a local strategy (fixations 4–9; short fixations, short saccades) after the first examination on the first object (left). In the current study, saccade amplitude increases after training for the experimental group for both cubes task (**a**) and the molecules task (**b**). The number of saccades between objects decreased after training for the experimental group for the cubes task (**c**) and the molecules task (**d**)

Saccade amplitude was averaged across the first three observations of the stimuli. Observations include all consecutive fixations on an object until a fixation is made outside the object or on the other object. For example, participants often started a trial by examining the left stimulus (first observation), switched to the right stimulus (second observation), and then switched back to the left stimulus (third observation; see Fig. 4). The average number of observations per trial was variable (pre-training *M* = 6.84, *SD* = 1.10; post-training *M* = 5.59, *SD* = 1.00), but participants always made at least three observations per trial. Saccades were only included in this analysis if they occurred within an observation of a given object; that is, only saccades between two consecutive fixations on the same object were included. Saccades between the two objects (or observations) were excluded for this analysis (see “Saccades between objects” subsections below). We conducted a 2 (training group) × 2 (session) × 10 (angular disparity) mixed factors ANOVA. We added angular disparity to these analyses to determine if increased task difficulty impacted eye movements. There was a significant main effect of angular disparity, *F*(9,306) = 7.98, *p* < .001, *η*^*2*^_*p*_ = .19, BF_10_ >  1000, in that saccade amplitude increased as angular disparity increased. There was a significant main effect of session, *F*(1,34) = 15.85, *p* < .001, *η*^*2*^_*p*_ = .32, BF_10_ = 49.49, in that saccade amplitudes were larger after training than before training (Fig. 4a). There was a significant interaction between angular disparity and session, *F*(9,306) = 2.96, *p* = .002, *η*^*2*^_*p*_ = .08, BF_10_ = 3.77. This interaction was driven by larger saccade amplitudes post-training than pre-training for all angular disparities (*p*_s_ < .036), except for 100 degrees of angular disparity (*p* = .15). Angular disparity did not significantly interact with any other variables (*p*_s_ > .25). There was no main effect of training condition, *F*(1,34) = 0.21, *p* = .65, *η*^*2*^_*p*_ = .01, BF_01_ = 0.37, and no interaction between training condition and session, *F*(1,34) = 0.454, *p* = .47, *η*^*2*^_*p*_ = .01, BF_01_ = 0.31. Follow-up tests revealed a significant increase in saccade amplitude after training for the experimental group, *t*(17) = 3.71, *p* = .002, BF_10_ = 26.42, but training did not impact saccade amplitude for the control group, *t*(17) = 1.99, *p* = .063, BF_10_ = 1.09. There was no significant difference between the two training groups before training, *t*(34) = 0.03, *p* = .98, BF_01_ = 0.32, or after training, *t*(34) = 0.59, *p* = .56, BF_01_ = 0.37. Overall, these results suggest that behavioral improvements in the experimental group may be due to more complete representational encoding.

#### Saccade amplitude: molecules task

There was a significant main effect of angular disparity, *F*(9,306) = 7.81, *p* < .001, *η*^*2*^_*p*_ = .19, BF_10_ >  1000, in that saccade amplitude increased as angular disparity increased. Angular disparity did not significantly interact with any other variables (*p*_s_ > .25). There was a significant main effect of session, *F*(1,34) = 7.79, *p* = .009, *η*^*2*^_*p*_ = .19, BF_10_ = 8.10, in that saccade amplitudes were larger after training than before training (Fig. 4b). There was also a significant interaction between angular disparity and session, *F*(9,306) = 13.97, *p* < .001, *η*^*2*^_*p*_ = .29, BF_10_ >  1000. This interaction was driven by larger saccade amplitudes post-training than pre-training for 20, 60, 100, 140, and 180 degrees of angular disparity (*p*_s_ < .001). Angular disparity did not significantly interact with any other variables (*p*_s_ > .27). There was no main effect of training condition, *F*(1,34) = 0.37, *p* = .55, *η*^*2*^_*p*_ = .01, BF_01_ = 0.49, and no interaction, *F*(1,34) = 0.01, *p* = .91, *η*^*2*^_*p*_ < .001, BF_01_ = 0.25. There was a significant increase in saccade amplitude after training for the experimental group, *t*(17) = 3.64, *p* = .002, BF_10_ = 13.94, but training did not impact saccade amplitude for the control group, *t*(17) = 1.54, *p* = .14, BF_01_ = 0.69. There were no differences in saccade amplitude for the two training conditions pre-training, *t*(34) = 0.58, *p* = .56, BF_01_ = 0.37, or post-training, *t*(34) = 0.59, *p* = .56, BF_01_ = 0.37. These results are similar to the saccade amplitude results for the cubes task. These results mimic those of the cubes task and suggest that training on a simple 3D mental rotation task may lead to encoding more complete representations of 3D molecules.

#### Saccades between objects: cubes task

The saccades between objects variable was calculated as the number of times participants made a saccade from one object to the other object. There was a significant main effect of angular disparity, *F*(9,306) = 59.23, *p* < .001, *η*^*2*^_*p*_ = .64, BF_10_ >  1000, in that the number of saccades between objects increased as angular disparity increased. There was a significant main effect of session, *F*(1,34) = 41.73, *p* = .001, *η*^*2*^_*p*_ = .55, BF_10_ >  1000, in that participants made more saccades between objects pre-training than post-training (Fig. 4c). There was also a significant interaction between angular disparity and session, *F*(9,306) = 6.48, *p* < .001, *η*^*2*^_*p*_ = .16, BF_10_ = 18.00. This interaction was driven by fewer saccades between objects post-training than pre-training for all angular disparities (*p*_s_ < .003). Angular disparity did not significantly interact with any other variables (*p*_s_ > .46). There was a significant main effect of training condition, *F*(1,34) = 4.41, *p* = .043, *η*^*2*^_*p*_ = .15, BF_10_ = 1.14, in that the control group made more saccades between objects than the experimental group. There was no interaction between session and training condition, *F*(1,34) = 1.39, *p* = .25, *η*^*2*^_*p*_ = .04, BF_01_ = 0.36. Follow-up tests revealed a significant decrease in the number of saccades between objects after training for the experimental group, *t*(17) = 4.99, *p* < .001, BF_10_ = 254.9, and the control group, *t*(17) = 4.01, *p* = .001, BF_10_ = 48.46. There were no differences in the number of saccades between objects for the two training conditions pre-training, *t*(34) = 0.97, *p* = .34, BF_01_ = 0.47, but post-training, the experimental group made significantly fewer saccades between objects than the control group, *t*(34) = 2.63, *p* = .013, BF_10_ = 4.14. Overall, these results suggest that, after training, participants could require fewer cycles through the encode–rotate–compare process, suggesting that they may have encoded a more complete representation.

#### Saccades between objects: molecules task

There was a significant main effect of angular disparity, *F*(9,306) = 28.06, *p* < .001*, η*^*2*^_*p*_*= .*45, BF_10_ >  1000, in that the number of saccades between objects increased as angular disparity increased. There was a significant main effect of session, *F*(1,34) = 11.35, *p* = .002, *η*^*2*^_*p*_ = .25, BF_10_ = 19.94, in that participants made more saccades between objects pre-training than post-training (Fig. 4d). There was also a significant interaction between angular disparity and session, *F*(9,306) = 5.22, *p* < .001, *η*^*2*^_*p*_ = .13, BF_10_ = 8.28. This interaction was driven by fewer saccades between object post-training than pre-training for 0, 20, 40, 60, 80, 120, and 160 degrees of angular disparity (*p*_s_ < .035). Angular disparity did not significantly interact with any other variables (*p*_s_ > .54). There was no main effect of training condition, *F*(1,34) = 0.88, *p* = .35, *η*^*2*^_*p*_ = .03, BF_01_ = 0.51, and no interaction, *F*(1,34) = 0.13, *p* = .86, *η*^*2*^_*p*_ = .001, BF_01_ = 0.25. There was no difference in the number of saccades between objects as a result of experimental training, *t*(17) = 2.04, *p* = .06, BF_10_ = 4.82, but the control training did result in a significant decrease in the number of saccades between objects, *t*(17) = 2.85, *p* = .01, BF_10_ = 1.30. There were no differences in the number of saccades between objects for the two training conditions pre-training, *t*(34) = 0.87, *p* = .39, BF_01_ = 0.43, or post-training, *t*(34) = 0.79, *p* = .43, BF_01_ = 0.41.

#### Dwell time slope: cubes task

We analyzed dwell time by calculating the slope of dwell time across the ten angular disparities. Dwell time was averaged across observations 2 and 3 in order to capture the rotation process. The results revealed no main effect of session, *F*(1,34) = 3.72, *p* = .06, *η*^*2*^_*p*_ = .10, BF_10_ = 1.01, or training condition, *F*(1,34) = 1.39, *p* = .25, *η*^*2*^_*p*_ = .04, BF_01_ = 0.51, and no interaction, *F*(1,34) = 0.48, *p* = .50, *η*^*2*^_*p*_ = .01, BF_01_ = 0.38. Overall, these results suggest that training on a mental rotation task may not be increasing the efficiency of the rotation process.

#### Dwell time slope: molecules task

The results revealed no main effect of session, *F*(1,34) = 1.07, *p* = .31, *η*^*2*^_*p*_ = .03, BF_01_ = 0.44, or training condition, *F*(1,34) = 1.64, *p* = .21, *η*^*2*^_*p*_ = .05, BF_01_ = 0.59, and no interaction, *F*(1,34) = 1.20, *p* = .28, *η*^*2*^_*p*_ = .03, BF_01_ = 0.64. These results are identical to the cubes task and suggest that the eye-tracking measures are revealing effects of mental rotation training on the completeness of the object representation but not on the rotation process.

### How does performance and object encoding change across training? (Sessions 2–7)

#### Experimental training: cubes task

##### Accuracy

**Fig. 5:**
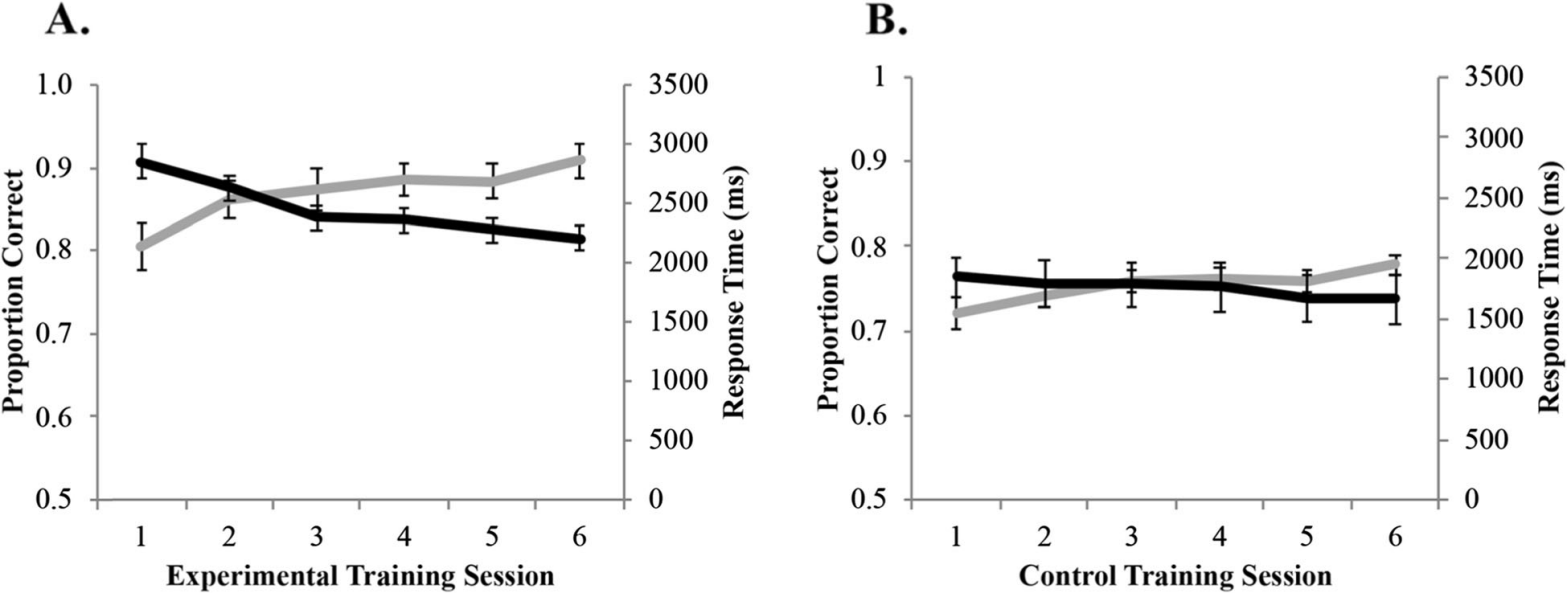
For the experimental training (**a**), accuracy (gray line) increased across training sessions and RT (black line) decreased. For the control training (**b**), accuracy (gray line) increased across training sessions and RT (black line) did not change

Session 2 data from one participant were lost. One-way ANOVA was conducted to examine the impact that the six training sessions had on accuracy during training (Fig. 5a), and the results revealed a significant effect of training session, *F*(5,80) = 18.92, *p* < .001, *η*^*2*^_*p*_ = .54, BF_10_ >  1000. To further investigate how many training sessions were needed to achieve this effect, we conducted paired samples *t* tests to compare each training session with the subsequent session (i.e., session 1 with session 2, session 2 with session 3, etc.). There were significant increases in accuracy from session 1 to session 2, *t*(17) = 3.50, *p* = .003, BF_10_ = 14.86, and from session 2 to session 3, *t*(17) = 3.34, *p* = .004, BF_10_ = 11.27. There were no differences between session 3 and session 4 (*p* = .27, BF_01_ = 0.43) or between session 4 and session 5 (*p* = .85, BF_01_ = 0.25), but there was another significant increase in accuracy from session 5 to session 6, *t*(17) = 2.49, *p* = .02, BF_10_ = 2.63. Overall, these results suggest that the greatest improvement during training occurs in the first three training sessions.

##### Response time

Session 2 data from one participant were lost. The results revealed a significant effect of training session (Fig. 5a), *F*(5,80) = 15.81, *p* < .001, *η*^*2*^_*p*_ = .50, BF_10_ >  1000. There were significant decreases in RT from session 1 to session 2, *t*(17) = 2.90, *p* = .011, BF_10_ = 5.16, and from session 2 to session 3, *t*(17) = 3.07, *p* = .007, BF_10_ = 6.96. There were no differences among sessions 4–6 (*p*_s_ > .15, BFs_10_ < 0.63). Overall, these results replicate the accuracy data and suggest that the greatest improvement during training occurs in the first three training sessions.

##### Saccade amplitude

Eye-tracking data for a given session were lost from three participants (one for session 1, one for session 2, and one for session 4). There was a significant effect of training session, *F*(5,70) = 3.87, *p* = .004, *η*^*2*^_*p*_ = .22, BF_10_ = 10.23, and significant increases in saccade amplitude from session 3 to session 4, *t*(17) = 2.75, *p* = .014, BF_10_ = 4.02. There were no other differences among consecutive sessions (*p*_s_ > .12, BFs_01_ < 0.75).

##### Saccades between objects

Eye-tracking data for a given session were lost from three participants (one for session 1, one for session 2, and one for session 4). There was a significant effect of training session, *F*(5,70) = 5.32, *p* < .001, *η*^*2*^_*p*_ = .28, BF_10_ = 84.52, and a significant decrease in the number of saccades between objects from session 1 to session 2, *t*(17) = 2.58, *p* = .02, BF_10_ = 3.00, and from session 2 to session 3, *t*(17) = 3.03, *p* = .008, BF_10_ = 6.46. There were no other differences among consecutive sessions (*p*_s_ > .23, BFs_01_ < 0.49). Dwell time slope was not analyzed, because there were no differences from pre- to post-training, as indicated above.

#### Control training: numerical estimation task

##### Accuracy

There was a significant effect of training session (Fig. 5b), *F*(5,85) = 5.74, *p* < .001, *η*^*2*^_*p*_ = .24, BF_10_ = 126.28, and significant increases in accuracy from session 1 to session 2, *t*(17) = 2.13, *p* = .048, BF_10_ = 1.50, and from session 5 to session 6, *t*(17) = 2.48, *p* = .024, BF_10_ = 2.57. There were no other significant differences in accuracy for the control task. These results demonstrate that the numerical estimation task was a sufficiently difficult, suitable control task and that training increased accuracy.

##### Response time

There was no significant effect of training session on RT, *F*(5,85) = 1.07, *p* = .38, *η*^*2*^_*p*_ = .06, BF_01_ = 0.14 (Fig. 5b).

### Brain imaging

#### Mental rotation activates different brain regions than not rotating

**Fig. 6:**
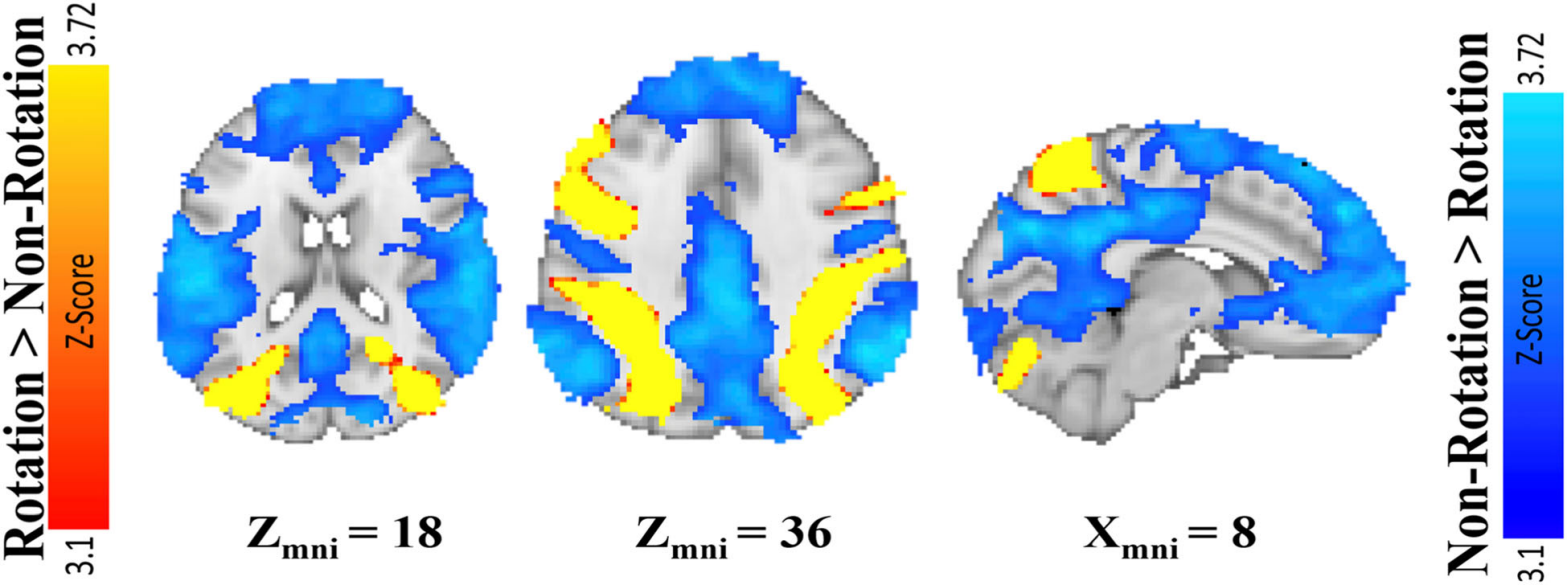
Rotation blocks (red/yellow) resulted in significantly greater activation in the visuospatial network than nonrotation blocks (blue), which activated the default mode network

A whole-brain analysis was conducted on the pre-training mental rotation task data to examine brain activation in areas that were more active during rotation blocks than during control blocks. The criteria for activation in the whole-brain analysis were set at an alpha threshold of *p* < .01 at the voxel level and corrected for multiple comparisons at the cluster level (*p* < .05). The results are reported in Fig. 6 and revealed robust VSN activation during rotation blocks including bilateral superior parietal lobes (see yellow areas in Fig. 6 and Table 2). We also observed greater activation in rotation blocks than in nonrotation blocks in bilateral M1 and premotor cortex, bilateral LOC, and bilateral anterior insula, as well as the left dorsolateral prefrontal cortex (DLPFC). On the other hand, we observed more robust activation of the default mode network during nonrotation than rotation blocks. This included activation of bilateral posterior cingulate cortex, medial prefrontal cortex, angular gyrus, medial temporal lobes, and frontal pole (see blue areas in Fig. 6 for details). Overall, these results replicate previous research (Carpenter et al., 1999; Halari et al., 2006; Hugdahl, Thomsen, & Ersland, 2006; Jordan, Heinze, Lutz, Kanowski, & Jäncke, 2001; Logie et al., 2011; Wraga, Shephard, Church, Inati, & Kosslyn, 2005) in that mentally rotating used distinct brain regions compared with not rotating the same stimuli.

**Table 2:** Areas of activation: mental rotation versus control task

Cluster no.	Cluster *k*	Region of local maxima	*Z*	*P* value	*H*	MNI		
						*x*	*y*	*z*
1	3935	Superior lateral occipital cortex	6.39	< .001	L	− 20	− 64	48
		Posterior supramarginal gyrus	4.83	< .001	L	− 34	− 48	52
		Anterior supramarginal gyrus	4.58	< .001	L	− 32	− 40	38
		Superior parietal lobule	4.55	< .001	L	− 32	− 56	66
2	3860	Superior lateral occipital cortex	5.35	< .001	R	20	− 64	66
		Superior parietal lobule	5.33	< .001	R	20	− 62	58
		Precuneus cortex	5.15	< .001	R	14	− 66	60
		Anterior supramarginal gyrus	5.1	< .001	R	48	− 34	48
3	996	Superior frontal gyrus	4.8	< .001	L	− 26	− 4	62
		Motor cortex	4.55	< .001	L	− 30	2	52
4	994	Motor cortex	5	< .001	R	30	2	68
		Precentral gyrus	4.21	< .001	R	30	− 8	62
5	347	Precentral gyrus	4.55	< .001	L	− 50	4	32
		Inferior frontal gyrus	3.81	< .001	L	− 34	6	28
6	327	Cerebellum	4.48	< .001	R	34	− 44	− 44
7	235	Cerebellum	5.34	< .001	L	− 18	− 56	− 44
8	187	Lateral occipital cortex	3.96	< .001	L	− 50	− 64	− 4
9	171	Occipital pole	4.26	< .001	L	− 34	− 92	− 8

*MNI* Montreal Neurological Institute

#### Relationship between brain activation, behavior, and eye movements

**Fig. 7:**
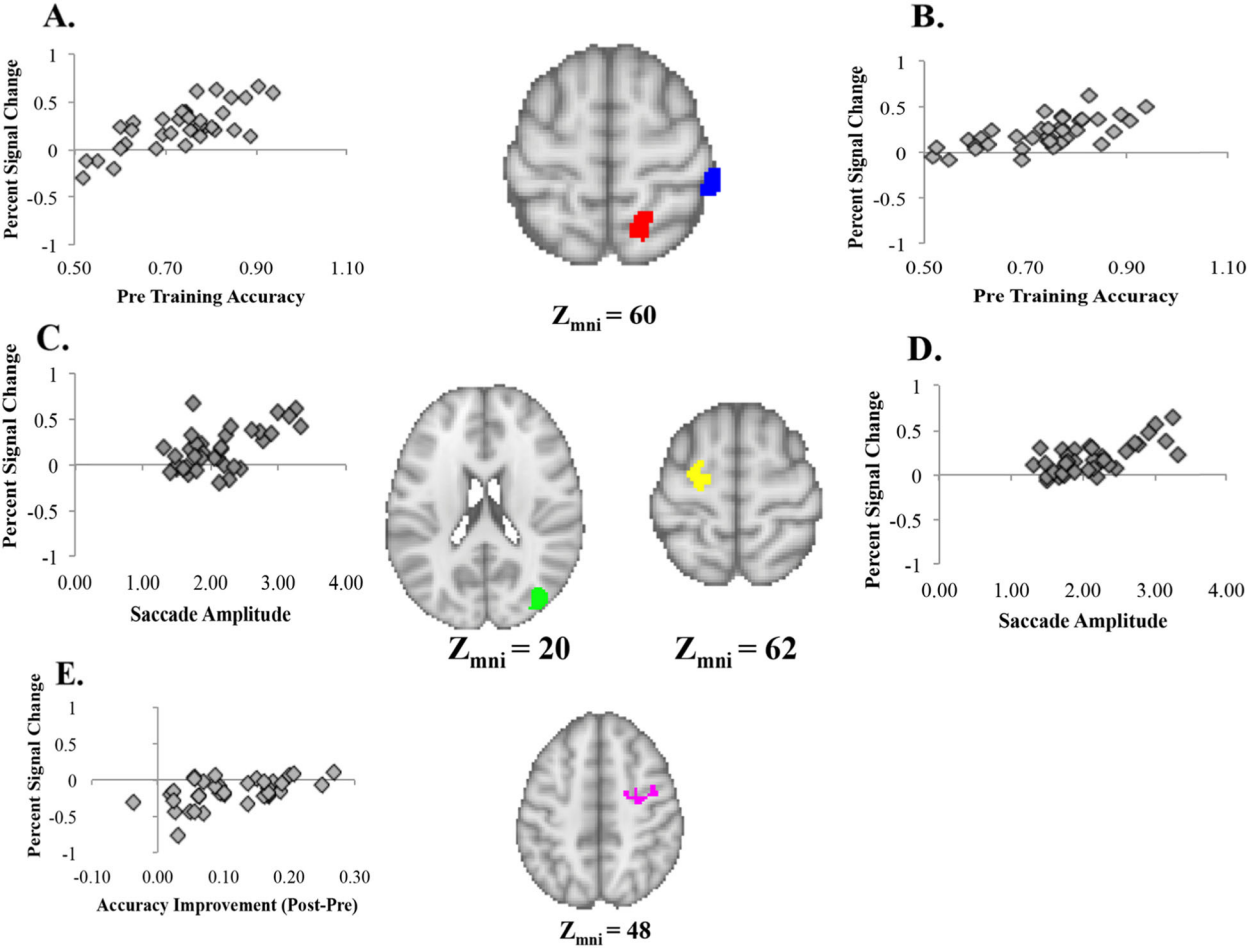
Several covariate analyses were conducted using pre-training brain activation data (session 2). Activation in the posterior lateral occipital cortex (**a**; red) and supramarginal gyrus (**b**; blue) significantly predicted pre-training accuracy (during session 1). Activation in the right lateral occipital cortex (**c**; green) and the left motor cortex (**d**; yellow) significantly predicted pre-training saccade amplitude during the first observation of an object. Activation in the right motor cortex (**e**; pink) significantly predicted accuracy improvement (session 9 accuracy − session 1 accuracy)

In order to examine the relationship between individual differences in brain activation and accuracy, we conducted a covariate analysis to determine if pre-training brain activation significantly predicted pre-training accuracy. We used accuracy during the first pre-training session for the cubes task and differential brain activation for rotation blocks compared with nonrotation blocks during the fMRI task. The results revealed two clusters related to the relationship between pre-training accuracy and pre-training brain activation during rotation blocks. These clusters corresponded to the right posterior aspects of the lateral occipital cortex (pLOC) (Fig. 7a) and the right supramarginal gyrus (Fig. 7b). Importantly, both of these areas are part of the VSN, described above. In order to visualize the effects, we extracted the percentage signal change from these clusters during the pre-training rotation blocks and plotted the percentage signal change along with pre-training accuracy (Fig. 7a and b). These results suggest that individuals who perform best on mental rotation tasks before training also tend to have the highest brain activation differential (i.e., rotation block – nonrotation block) in the right supramarginal gyrus and the right pLOC. Given the role of the LOC in encoding representations of multiple parts and the spatial relationships among the parts (Erdogan et al., 2016), this supports the eye-tracking data above in suggesting that encoding the spatial relationships between object parts leads to higher mental rotation accuracy.

A second covariate analysis was used to determine if pre-training brain activation significantly predicted saccade amplitude during the first observation of an object. We conducted covariate analyses for accuracy improvement and brain activation during the control and rotation blocks separately. For rotation blocks, the results revealed two clusters where pre-training saccade amplitude during the first observation of an object related to pre-training brain activation. These clusters corresponded to the right pLOC (Fig. 7c) and the left primary motor cortex (Fig. 7d). Extracted percentage signal change revealed a positive relationship between pre-training saccade amplitude during the first observation of an object and activation in the left primary motor cortex and the right LOC. These results suggest that individuals who have longer saccades while encoding the first object also tend to have the highest brain activation in the left primary motor cortex and the right ventral LOC. These results may suggest that more complete representations composed of parts and the spatial relationships between the parts (Erdogan et al., 2016), characterized by longer saccades, are associated with greater ventral LOC activation. Additionally, the relationship between saccade amplitude and motor cortex may suggest that longer saccades are also associated with the actual rotation process.

We conducted an additional covariate analysis to determine if pre-training brain activation significantly predicted accuracy improvement. We calculated accuracy improvement by subtracting accuracy during the first pre-training session for the cubes task from accuracy during the post-training cubes task. For rotation control blocks, there were no clusters that significantly predicted accuracy improvement. However, activation in the right primary motor cortex during control rotation blocks significantly predicted accuracy improvement (Fig. 7e). Extracted percentage signal change revealed a positive correlation between accuracy improvement and differential activation in the right primary motor cortex. These results suggest that individuals who improve the most from training, regardless of training condition, have reduced motor area inhibition during nonrotation control blocks. Overall, these results suggest that using motor areas, even when mental rotation is not necessary, leads to the greater improvement in accuracy.

#### Impact of training on brain activation

**Fig. 8:**
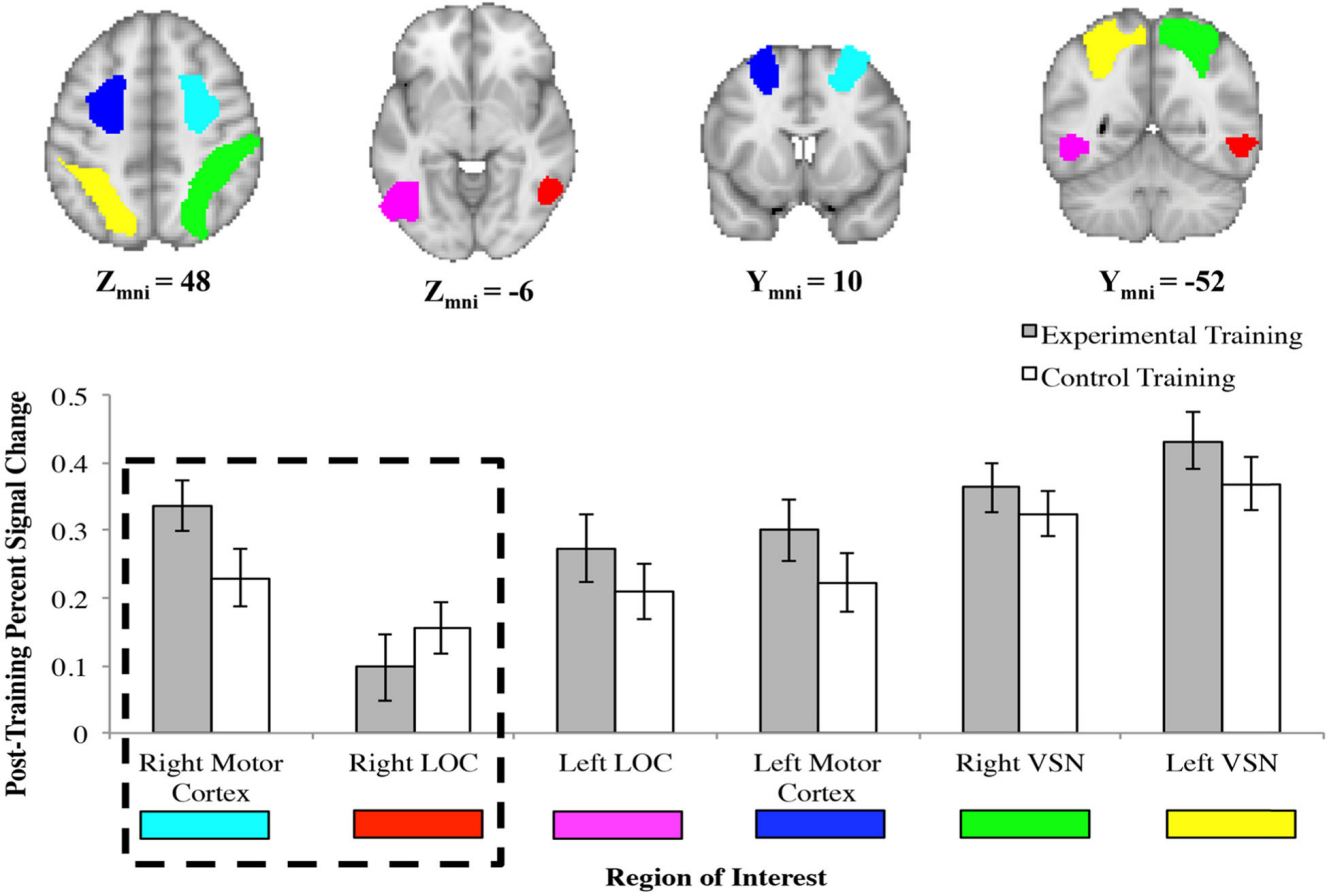
Masks were created from rotation blocks during pre-training (session 2) for the visuospatial network (yellow and green), lateral occipital cortex (pink and red), and motor areas (blue and cyan). We used these regions to conduct logistic regression to determine which brain areas could predict training group assignment. The dashed box indicates the two brain regions that significantly predicted group assignment

Masks were created from the pre-training scan of the left and right VSN and eroded by 4 mm (to restrict our mask to a more precise location and to better capture peak activation; Fig. 8, green and yellow). Masks were also created for the left and right LOC (Fig. 8, pink and red) and motor cortices (Fig. 8, blue and cyan).

Given that mental rotation involves multiple processes, most notably encoding and rotation, we were interested in the possibility that training impacts the network of brain regions associated with mental rotation rather than any single region alone. Therefore, we conducted a backward stepwise logistic regression in order to determine if post-training brain activation (Fig. 8) could predict training condition. We used the left and right VSN, left and right LOC, and left and right motor cortices as predictors (Table 3). The model achieved statistical significance on the fifth step, χ^2^ (1) = 6.54, *p* = .038, which included the right motor cortex (*R*^2^ = .21, *p* = .03) and right LOC (*R*^2^ = .12, *p* = .10) and was 69.4% accurate in predicting which participants belonged to the experimental group (and control group).

**Table 3:** Backward stepwise logistic regression analysis on right lateral occipital cortex and right motor areas predicting training group

Model	Predictor	*P* value	Odds ratio (95% CI)
Step 1	Left VSN	.86	0.35 (0, > 1000)
	Right VSN	.56	47.87 (0, > 1000
	Left LOC	.33	0.04 (0, 26.91)
	Right LOC	.15	50.23 (0.23, > 1000)
	Left Motor	.43	37.88 (0.004, > 1000)
	Right Motor	.06	0 (0, 1.55)
Step 2	Right VSN	.57	26.41 (0, > 1000)
	Left LOC	.25	0.03 (0, 11.38)
	Right LOC	.14	55.30 (0.28, > 1000)
	Left Motor	.44	28.37 (0.01, > 1000)
	Right Motor	.06	0 (0, 1.57)
Step 3	Left LOC	.31	0.06 (0, 12.57)
	Right LOC	.07	98.86 (0.68, > 1000)
	Left Motor	.35	46.90 (0.01, > 1000)
	Right Motor	.07	0 (0, 1.66)
Step 4	Left LOC	.49	0.19 (0.002, 20.12)
	Right LOC	.09	71.20 (0.56, > 1000)
	Right Motor	.06	0.01 (0, 1.26)
Step 5	Right LOC	.10	41.00 (0.48, > 1000)
	Right Motor	.03	0.004 (0, 0.63)

*Abbreviations: CI* confidence interval, *LOC* lateral occipital cortex, *VSN* visuospatial network

Finally, we conducted independent samples *t* tests to compare the two training groups post-training for each region of interest (ROI). The results revealed no significant differences between training groups for any ROI (see Table 4 for a summary of the results). The results of this logistic regression in combination with the null *t* test results suggest that no one brain area improves as a result of mental rotation training; rather, there is improvement in a network of brain regions associated with mental rotation ability, most notably in the right motor cortex and LOC.

**Table 4:** Independent samples *t* tests: difference in activation between training groups post-training for each region of interest

Region of interest	*t*	*P* value	Mean difference (95% CI)	BF_10_
Left VSN	1.16	.25	0.06 (− 0.05, 0.17)	< 1
Right VSN	0.79	.44	0.04 (− 0.06, 0.14)	< 1
Left LOC	0.23	.82	0.01 (− 0.12, 0.15)	< 1
Right LOC	0.93	.36	− 0.06 (− 0.19, 0.07)	< 1
Left Motor Cortex	1.24	.22	0.07 (− 0.05, 0.20)	1.24
Right Motor Cortex	1.88	.07	0.11 (− 0.01, 0.22)	< 1

*Abbreviations: BF* Bayes factor, *CI* confidence interval, *LOC* lateral occipital cortex, *VSN* visuospatial network

**Fig. 9:**
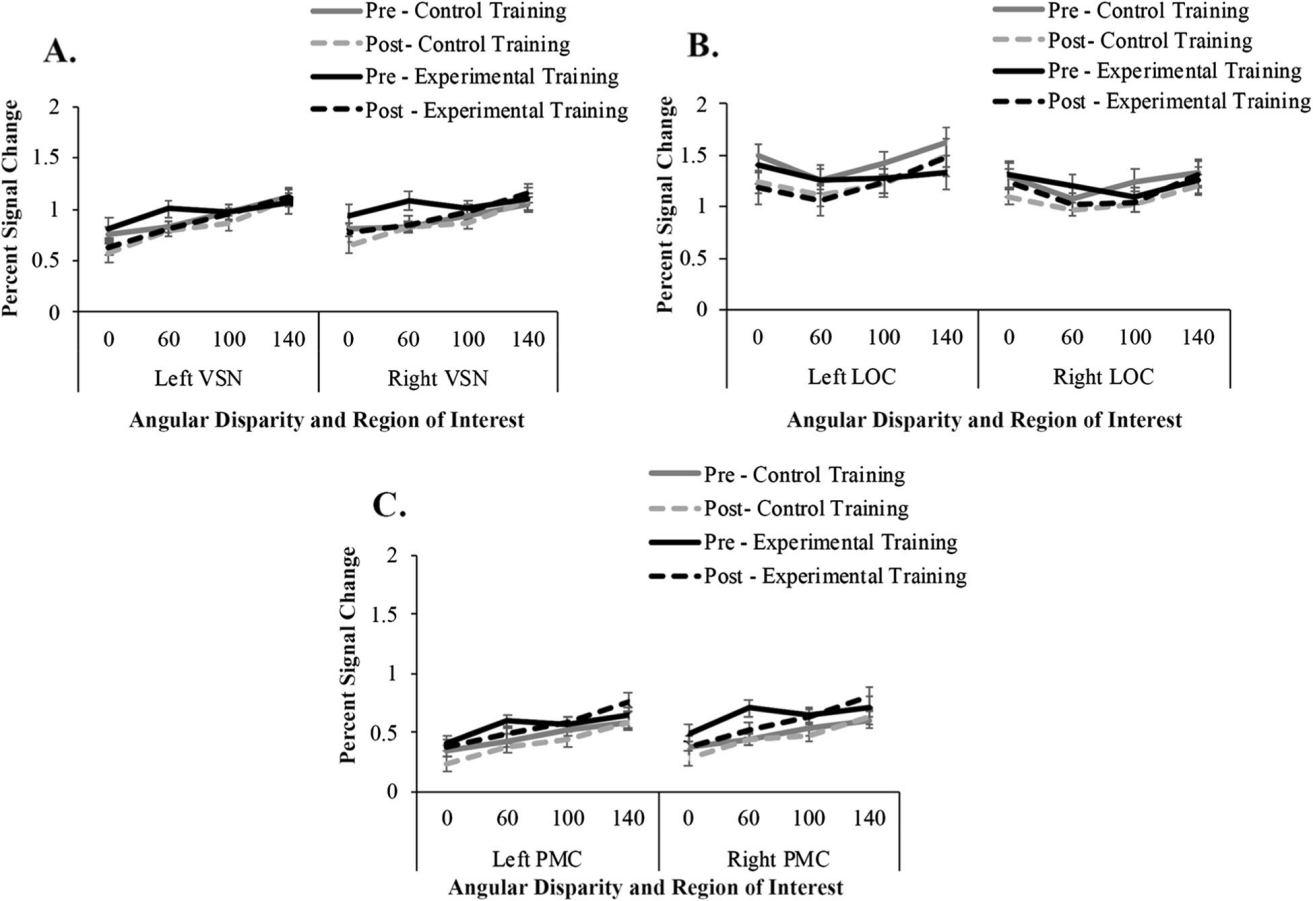
Brain activation in the visuospatial network (**a**), lateral occipital cortex (**b**), and motor cortex (**c**) as a function of angular disparity. Error bars represented standard error

Next, we sought to determine which regions increased in activity as a function of the linear increase in rotation angle. We modeled the fMRI data separately for each angular disparity (0 [control blocks], 60, 100, 140) and used a linear mean-centered parameter to examine the impact of angular disparity on brain activation. Values significantly greater than zero would indicate that brain activation increased as angular disparity increased. Visualizations of these parameters are presented in Fig. 9. We conducted one-sample *t* tests to test the parameter against baseline pre- and post-training. Pre-training, the angular disparity parameter was significantly different from zero for the left and right VSN and the left and right motor cortex. Post-training, the angular disparity parameter was significantly different from zero for the left and right VSN, the left and right motor cortex, and left LOC (see Table 5 for results summary). In order to determine if angular disparity impacted brain activation differently based on training condition, we also conducted a repeated measures ANOVA for each ROI separately. There was a main effect of session in that the angular disparity parameter was larger after training than before training for the left and right VSN, the left LOC, and the left and right motor cortices. There was no main effect of session for the right LOC, nor were there any main effects of training condition and no interactions (see Table 6 for results summary). Overall, these results replicate previous research (Carpenter et al., 1999; Cohen et al., 1996; Gogos et al., 2010) in that brain activation increases as angular disparity increases. Specifically, the linear increase in brain activation as a function of angular disparity suggests that changes in activation in the motor cortices and LOC pre- to post-training are due to an improvement in the rotation process. Furthermore, the right motor cortex and right LOC were the best predictors of training group in the logistic regression. On the basis of our findings, the experimental group’s behavioral improvements in mental rotation ability are likely due to the combination of improved rotation (VSN, motor cortex) and encoding more detailed representations (LOC, saccade amplitude).

**Table 5:** One-sample *t* tests: activation as a function of angular disparity for each region of interest

Region of interest	Session	*t*	*P* value	Mean difference (95% CI)	BF_10_
Left VSN	Pre-training	6.03	< .001	0.26 (0.17, 0.34)	> 1000
	Post-training	9.82	< .001	0.40 (0.42, 0.48	> 1000
Right VSN	Pre-training	4.76	< .001	0.17 (0.10, 0.24)	684.1
	Post-training	8.41	< .001	0.29 (0.22, 0.37)	> 1000
Left LOC	Pre-training	0.47	.64	0.02 (−0.07, 0.11)	< 1
	Post-training	3.71	.001	0.18 (0.08, 0.27)	42.37
Right LOC	Pre-training	− 0.03	.98	− 0.001 (− 0.08, 0.07)	< 1
	Post-training	− 0.29	.78	− 0.01 (− 0.11, 0.09)	< 1
Left motor cortex	Pre-training	5.25	< .001	0.20 (0.12, 0.28)	> 1000
	Post-training	7.15	< .001	0.29 (0.21, 0.37)	> 1000
Right motor cortex	Pre-training	4.57	< .001	0.19 (0.11, 0.28)	408.6
	Post-training	7.18	< .001	0.30 (0.22, 0.39)	> 1000

*Abbreviations: BF* Bayes factor, *CI* confidence interval, *LOC* lateral occipital cortex, *VSN* visuospatial network

**Table 6:** Mixed factor analysis of variance (session × training): impact of angular disparity on activation for each region of interest

Region of interest	Effect	*F*	*P* value	*η*^*2*^_*p*_	BF_10_
Left VSN	Session	12.74	.001	.27	> 1000
	Training	0.33	.57	.01	< 1
	Interaction	1.58	.22	.04	< 1
Right VSN	Session	10.76	.002	.24	> 1000
	Training	0.40	.53	.01	< 1
	Interaction	0.47	.50	.01	< 1
Left LOC	Session	6.13	.02	.15	109.4
	Training	0.39	.54	.01	< 1
	Interaction	3.34	.08	.09	1.26
Right LOC	Session	0.04	.84	.001	< 1
	Training	0.43	.52	.01	< 1
	Interaction	0.004	.95	< .001	< 1
Left motor cortex	Session	4.31	.045	.11	< 1
	Training	0.05	.82	.002	< 1
	Interaction	0.53	.47	.02	< 1
Right motor cortex	Session	6.68	.01	.16	19.8
	Training	0.07	.79	.002	< 1
	Interaction	1.77	.19	.05	< 1

*Abbreviations: BF* Bayes factor, *LOC* lateral occipital cortex, *VSN* visuospatial network

## Discussion

The current study used a multimethod approach to examine the neurocognitive mechanisms associated with mental rotation skill improvement. We used eye-tracking and fMRI to determine changes in attention allocation and neural activity associated with improvements from training to help determine the roles of (1) encoding a complete representation and (2) the ability to mentally rotate the representation. The current study is a novel contribution to the field of spatial reasoning research because of the multimethod design and our strict adherence to the guidelines for an optimal training study (see Uttal et al., 2013, for review). We measured behavior, eye movements, and brain activation in order to define the attentional and neurological changes associated with mental rotation training. Specifically, we were interested in how mental rotation impacted encoding and rotating mental representations of 3D stimuli. We demonstrated that behavioral improvements in mental rotation ability are likely due to the combination of improved rotation (VSN, motor cortex) and encoding more detailed representations (LOC, saccade amplitude).

The current study replicated several previous studies (Leone, Taine & Droulez, 1993; Meneghetti et al., 2017; Provost, Johnson, Karayanidis, Brown & Heathcote, 2013; Rodán et al., 2016; Wright et al., 2008) which found that training on a mental rotation task improves performance on that same task. The question that is less certain from the previous literature is when training on one task can transfer to other tasks or other stimuli (Uttal et al., 2013). The goal of the current study was to examine the possibility of near transfer (transfer to a new category of stimuli in an analogous spatial reasoning task). Other studies have examined how mental rotation skills transfer to other tasks such as spatial paper folding (Wright et al., 2008), perspective tests (Meneghetti et al., 2016, Meneghetti et al., 2017), or measures of working memory capacity (Rodán et al., 2016) with varying results, leaving conclusions uncertain. Therefore, we used an identical same/different task procedure for the trained (cubes task) and untrained stimulus categories (molecules task) to determine the degree of near transfer. The current study also differed from previous mental rotation studies by using a control task that mirrored the mental rotation training task except for the need for mental rotation to complete the task. Contrary to previous studies that have used control groups with tasks such as crossword puzzles (Meneghetti et al., 2016), personality questionnaires (Meneghetti et al., 2017), or word comparisons (Wright et al., 2008), our control group provides a better control for determining the effects of practicing mental rotation skills (see Simons et al., 2016). Finally, the use of a multimethod design is the other primary difference between the current study and previous mental rotation training studies. The current study is the first study to determine the extent of mental rotation skill development by comparing behavioral, oculomotor, and neurological results.

### Encoding and rotating representations

One of the goals of the current study was to determine whether mental rotation training leads to encoding more complete mental representations and/or more effective rotation of the mental representations. More complete encoding leads to longer saccade amplitudes (Davitt et al., 2014), and in the current study, we demonstrated a significant increase in saccade amplitude from pre- to post-training for the experimental group in the cubes and molecules tasks. Representations are more complete when they include more than a single part of an object, such as a whole-object representation from multiple viewpoints or multiple part-based representations that include the spatial relationships among the parts (Erdogan et al., 2016). Eye-tracking data from the current study suggest that the number of saccades between objects decreases as a result of training, which suggests that participants were encoding more complete representations, requiring fewer iterations of the encode–rotate–compare process (Larsen, 2014). However, it is important to note that participants still required about five saccades between objects, on average, before making a response, which suggests that participants were not using a holistic encoding process (e.g., encoding a representation of the whole object). Instead, it is more likely that mental rotation training led participants to encode the spatial relationship among object parts, leading to more complete representations.

To complement the encoding effects found in saccade amplitude, we also looked toward neural markers of encoding object representations. Increased LOC activation is also associated with encoding object parts and the spatial relationships among the parts (Erdogan et al., 2016). In the current study, post-training LOC activation was a predictor in the logistic regression used to predict training group. Additionally, we observed that increased pLOC pre-training was associated with higher pre-training accuracy. pLOC was also positively associated with saccade amplitude during the first observation of a stimulus. Specifically, participants who made longer saccades pre-training also had the most pLOC activation pre-training. These results may suggest that participants who tend to encode multiple object parts and their spatial relationships have higher mental rotation accuracy and greater pLOC activation before training. Overall, results of eye-tracking and fMRI suggest that encoding multiple parts of the objects and the spatial relationships among the parts leads to increased saccade amplitude, a decrease in the number of saccades between objects, and changes LOC activity.

In addition to encoding more complete representations, mental rotation training may also improve rotation of the representation. Traditionally, changes in representational rotation would be measured with the RT by angle of disparity slope. Although in the current study we found no changes in the RT slope as a function of training, rotation was also associated with motor cortex activation, which was an important ROI in several analyses in the current study. Specifically, we found that motor cortex activation (as a function of angular disparity) increased after training. Importantly, these findings were not specific to the experimental training condition. We also found that brain activation in motor areas for control rotation blocks was positively associated with accuracy improvement. These results suggest that using motor areas, even when mental rotation is not necessary (i.e., control blocks), leads to greater improvement in accuracy.

In the current study, the VSN, namely the superior parietal lobes, was more active during rotation blocks than nonrotation control blocks. Previous research using simultaneous fMRI and transcranial magnetic stimulation (TMS) has revealed that when TMS is used to inhibit activation in the superior parietal lobe, 2D mental rotation RTs were slower than without TMS (Graaf, Roebroeck, Goebel, & Sack, 2010; Sack et al., 2007). These results suggest that, in addition to motor areas, the VSN may also be engaged while rotating mental representations. Indeed, we found that VSN activation increased as a function of angular disparity. Overall, these results suggest that both the VSN and motor areas contribute to the rotation process.

Previous research has suggested that mental rotation performance improves with training because of a more efficient process of comparing the encoded and rotated representation of one stimulus with the other stimulus (Heil & Jansen-Osmann, 2008). An improvement in the comparison process may be evident by shorter fixation durations after the first object has been encoded. Furthermore, activity in prefrontal regions may change as the comparison process becomes more efficient. DLPFC activation during rotation tasks may signify the role of the visuospatial working memory system needed for the comparison process (Carpenter et al., 1999). However, our analyses did not reveal any areas in the DLPFC associated with mental rotation. We also analyzed fixation durations after the first object was encoded and found no differences as a result of training.

Overall, the results of the current study suggest that mental rotation training has the greatest impact on the encoding process and leads participants to encode more complete mental representation, including the spatial relationship among object parts. Furthermore, the results of the current study also highlight the importance of representation rotation, specifically with regard to the motor cortex. Specifically, activation in the LOC and motor areas made unique contributions to determining whether an individual completed mental rotation training or control training and suggested that mental rotation ability is associated with a network of brain regions rather than a specific brain area.

### Generalizability

We found evidence that participants were able to improve mental rotation performance on novel stimuli from the trained category of stimuli. In addition, we found evidence that these effects can generalize to a new category of stimuli. Individuals may have a general strategy for completing mental rotation tasks (e.g., analytic versus holistic), and they tend to use the same strategy on different mental rotation tasks (Janssen & Geiser, 2010). Therefore, the current results may be due to learning a more effective strategy for the mental rotation task with 3D cubes and then applying this strategy to a novel set of stimuli (e.g., 3D ball-and-stick molecular structures). The eye-tracking and neuroimaging data suggest that this strategy involved encoding a more complete representation that included multiple parts and their spatial relationships.

The generalization effects to the nontrained category were not large effects, but the BFs suggest that there is evidence that the experimental group had increased accuracy on the molecules task following training. The lack of a stronger generalization effect may have been due in part to the difficulty of the molecules mental rotation task. Accuracy was near chance for pre-training in the molecules task, suggesting that participants were not able to do the task. The shallow slope for the RT by angle of disparity function during pre-training suggests that participants were not rotating encoded representations. This may be because participants were unable to encode representations that they could use to do the rotation. Post-training, when object encoding had improved as evidenced by longer saccade amplitudes, rotation was possible, as evidenced by the increased slope and similarity of slope between molecules and cubes for the experimental group post-training.

Another possibility regarding the small generalization effect may be the training structure. Some training studies (Park & Brannon, 2014) have used an adaptable training program, where the task increases in difficulty as performance improves. It is possible that the experimental group training could have had a larger impact on the molecules task if training were adaptable based on performance. Overall, results of the current study suggest that the ability to encode more complete representations of stimuli may lead to improved mental rotation ability. However, an adaptable training program or less complex stimuli may be necessary to observe stronger effects.

### Implications for STEM disciplines

Mental rotation performance, as a measure of spatial reasoning skills, is well established to be a strong indicator of success in STEM disciplines (Wai et al., 2009) and in several professions, including chemistry (Bodner & Guay, 1997; Harle & Towns, 2011), engineering (Samsudin et al., 2011; Sorby, 2009), surgery (Stransky et al., 2010), and aviation (Dror et al., 1993). Understanding how to best train spatial reasoning depends on understanding the cognitive processing necessary to complete the task and capacity to improve each type of cognitive process involved. The current study shows that improvement in spatial reasoning performance relies on the ability to learn to encode multiple object parts of an object and the spatial relationships between the parts as well as the ability to engage motor areas. This knowledge will be useful in developing training protocols and improving spatial skills for STEM students. Ultimately, this has the potential for improved STEM retention and improved STEM performance, which could lead to an increase in the quantity, quality, and diversity of graduates in these disciplines. The current study demonstrated that mental rotation training with simple 3D cubes leads to increased accuracy for novel stimuli from the trained category, as well as, to some extent, for an untrained category, and impacts activation in the LOC, VSN, and motor areas.

The current study speaks not only to training mental rotation for improvements in STEM disciplines but also to the larger question of the effectiveness of brain training in general. Brain training programs are commonplace but not always supported by valid scientific evidence (see Simons et al., 2016, for review). Training for some skills may be limited in that there may be near transfer but not broad transfer. (Training on one spatial reasoning task may not improve performance on a different spatial reasoning task.) The degree to which a training effect has near or broad transfer may depend on the extent to which the training improves cognitive processing that is used in other related tasks. The current study provides a methodology (combining eye-tracking and fMRI with an optimal training protocol) that can be used to guide future research on the effectiveness of training and to produce more valid training programs. For example, results of the current study revealed that three training sessions is a sufficient amount of training to elicit behavioral and oculomotor differences between training groups. Training programs have the potential to optimize individuals’ performance in different disciplines, disorders, and across development (especially with aging); however, it is imperative that the programs are scientifically supported and developed on the basis of scientific evidence.

## Conclusion

Given that only about half of students who enter STEM undergraduate degree programs complete a STEM degree (Higher Education Research Institute, 2010) and that many are leaving because they lack the skills needed to be successful (Chen, 2013), it is important to determine if and how skills can be improved. Though mental rotation performance can be reliably measured and even improved, the extent of generalization and the role of cognitive processes in improvements in mental rotation performance are not well understood. We used eye-tracking and fMRI to determine changes in attention allocation and neural activity associated with improvements due to training to help determine the roles of (1) encoding a complete representation and (2) the ability to mentally rotate the representation. Improvements and performance can occur as a result of more complete encoding and more efficient rotation. Specifically, mental rotation improvement is associated with activity in a network of brain regions, most notably in the right motor cortex and LOC, suggesting that training programs aimed at improving mental rotation performance will need to simultaneously target both of these cognitive processes.

## Data Availability

The stimuli and datasets used during the current study are available from the corresponding author on reasonable request.

## Abbreviations

ANOVA: Analysis of variance
BF: Bayes factor
DLPFC: Dorsolateral prefrontal cortex
FILM: Oxford Centre for Functional Magnetic Resonance Imaging of the Brain Improved Linear Model
FLAME: Oxford Centre for Functional Magnetic Resonance Imaging of the Brain Local Analysis of Mixed Effects
fMRI: Functional magnetic resonance imaging
FMRIB: Oxford Centre for Functional Magnetic Resonance Imaging of the Brain
FOV: Field of view
GPA: Grade point average
LOC: Lateral occipital cortex
MCFLIRT: Motion correction using Oxford Centre for Functional Magnetic Resonance Imaging of the Brain linear image registration tool
pLOC: Posterior aspects of the lateral occipital cortex
ROI: Region of interest
RT: Response time
STEM: Science, technology, engineering, and mathematics
TE: Echo time
TMS: Transcranial magnetic stimulation
TR: Repetition time
VSN: Visuospatial network
